# CsIVP functions in vasculature development and downy mildew resistance in cucumber

**DOI:** 10.1371/journal.pbio.3000671

**Published:** 2020-03-23

**Authors:** Shuangshuang Yan, Kang Ning, Zhongyi Wang, Xiaofeng Liu, Yanting Zhong, Lian Ding, Hailing Zi, Zhihua Cheng, Xuexian Li, Hongyan Shan, Qingyang Lv, Laixin Luo, Renyi Liu, Liying Yan, Zhaoyang Zhou, William John Lucas, Xiaolan Zhang

**Affiliations:** 1 State Key Laboratories of Agrobiotechnology, Beijing Key Laboratory of Growth and Developmental Regulation for Protected Vegetable Crops, MOE Joint Laboratory for International Cooperation in Crop Molecular Breeding, China Agricultural University, Beijing, China; 2 Key Laboratory of Biology and Genetic Improvement of Horticultural Crops (South China), Ministry of Agriculture and Rural Affairs, College of Horticulture, South China Agricultural University, Guangzhou, China; 3 Department of Plant Nutrition, the Key Laboratory of Plant-Soil Interactions, China Agricultural University, Beijing, China; 4 Shanghai Center for Plant Stress Biology, Shanghai Institutes for Biological Sciences, Chinese Academy of Sciences, Shanghai, China; 5 State Key Laboratory of Systematic and Evolutionary Botany, Institute of Botany, Chinese Academy of Sciences, Beijing, China; 6 Department of Plant Pathology, China Agricultural University, Beijing, China; 7 College of Horticulture, and FAFU-UCR Joint Center for Horticultural Biology and Metabolomics, Haixia Institute of Science and Technology, Fujian Agriculture and Forestry University, Fuzhou, China; 8 College of Horticulture Science and Technology, Hebei Normal University of Science & Technology, Qinhuangdao, China; 9 Department of Plant Biology, University of California, Davis, California, United States of America; Cornell University, UNITED STATES

## Abstract

Domesticated crops with high yield and quality are frequently susceptible to pathogen attack, whereas enhancement of disease resistance generally compromises crop yield. The underlying mechanisms of how plant development and disease resistance are coordinately programed remain elusive. Here, we showed that the basic Helix-Loop-Helix (bHLH) transcription factor *C**ucumis* *s**ativus* *I**rregular* *V**asculature* *P**atterning* (*CsIVP*) was highly expressed in cucumber vascular tissues. Knockdown of *CsIVP* caused severe vasculature disorganization and abnormal organ morphogenesis. CsIVP directly binds to vascular-related regulators *YABBY5* (*CsYAB5*), *BREVIPEDICELLUS* (*CsBP*), and *AUXIN/INDOLEACETIC ACIDS4* (*CsAUX4*) and promotes their expression. Knockdown of *CsYAB5* resulted in similar phenotypes as *CsIVP*-RNA interference (RNAi) plants, including disturbed vascular configuration and abnormal organ morphology. Meanwhile, *CsIVP*-RNAi plants were more resistant to downy mildew and accumulated more salicylic acid (SA). CsIVP physically interacts with NIM1-INTERACTING1 (CsNIMIN1), a negative regulator in the SA signaling pathway. Thus, *CsIVP* is a novel vasculature regulator functioning in CsYAB5-mediated organ morphogenesis and SA-mediated downy mildew resistance in cucumber.

## Abstract 2

The novel vascular patterning regulator CsIVP functions to integrate the programming of organ morphogenesis and salicylic acid-mediated resistance to downy mildew in cucumber.

## Introduction

Crop domestication is a fundamental process promoting agriculture and human societal development. Compared with their wild ancestors, cultivated crops have features such as high yield and better quality, largely resulting from increased organ size, improved nutritional quality, synchronous ripening, and reduced dispersibility [1,2]. However, due to artificial selection, some stress-resistant traits in wild species were lost during domestication, and many cultivated varieties—with superior agronomic characteristics—are susceptible to disease and abiotic stresses [3]. On the other hand, for disease-resistant genotypes, plants often exhibit reduced yield potential and negative developmental attributes [4]. Furthermore, when plants are infected by pathogens, defense responses frequently occur with a cost of reduction in growth and reproduction [5]. In spite of intensive investigations into the basis of domestication [3,6,7], the underlying mechanism for co-regulation of development and disease resistance remains unclear.

The evolution of the plant vasculature system played a pivotal role in adaptation to various terrestrial environments. In contrast to nonvascular bryophytes, vascular plants underwent further organ differentiation and enlargement of plant stature, and enhanced resilience to environmental stress [8]. With evolutionary progression, collateral or bicollateral vascular systems became dominant in the angiosperms. *Arabidopsis* is an example of a collateral vascular arrangement, as its phloem is positioned in parallel on the single side of the xylem, whereas *Cucurbit* serves as a bicollateral vascular model, with its phloem on both sides of the xylem [9,10].

The plant vascular system is responsible for delivering resources throughout the plant [8]. Xylem carries water and mineral nutrients from the root to the shoot, whereas phloem transports photosynthetic products and various signals, from the source to sink organs [11]. The NAM/ATAF/CUC (NAC) transcription factors—VASCULAR-RELATED NAC-DOMAIN 7 (VND7) and VND6—function as major transcriptional switches that regulate myeloblastosis (MYB)-domain transcription factors (*MYBs*) and *IRREGULAR XYLEM* (*IRX*) family genes to control differentiation of tracheary elements in xylem tissues [12,13]. *KNOTTED1-LIKE HOMEOBOX GENE* 7 (*KNAT7*) and *BREVIPEDICELLUS* (*BP*) are involved in xylem differentiation by mediating biosynthesis of cell wall components [13,14]. Phloem differentiation is regulated by transcription factors, including *ALTERED PHLOEM DEVELOPMENT* (*APL*) and *OCTOPUS* (*OPS*) [15,16].

Vasculature patterning also reflects leaf morphogenesis [17,18]. The vein system outlines leaf shape; the xylem and phloem in leaf veins is correlated with leaf dorsiventrality, with phloem in the abaxial and xylem adaxial position [19]. Loss of function of transcriptional factors—including *PHABULOSA* (*PHB*), *KANADI* (*KAN*), or *YABBY* (*YAB*)—resulted in unifacial leaves, typically with only adaxial or abaxial epidermal identity, and aberrant xylem and phloem patterning in the vein [20,21]. In addition, the vasculature also participates in defense-related processes by transporting hormones, proteins, and RNAs [22]. However, no vascular regulator has been shown yet to mediate stress resistance directly.

Salicylic acid (SA) plays an important role in promoting the local defense against biotrophic and hemibiotrophic pathogens [23]. In the SA signaling pathway, NONEXPRESSOR OF PATHOGENESIS-RELATED GENES 1 (NPR1) acts as a transcriptional co-activator to activate *PATHOGENESIS-RELATED PROTEIN1* (*PR1*) and several transcription factors harboring the highly conserved WRKYGQK motif (*WRKY*), through interacting with the TGA family genes [24]. NIM1-INTERACTING1 (NIMIN1) appears to be a negative regulator in SA/NPR1 signaling, which functions through forming a ternary complex with NPR1 and a TGA factor that binds to the *PR-1* promoter [25]. Allocation of SA to defense inevitably reduces plant growth and reproduction [26], but the underlying mechanism remains largely unknown.

In this study, we identified a cucumber (*Cucumis sativus* L.) vasculature regulator, *Cucumis sativus* Irregular Vasculature Patterning (CsIVP), which functions in integrating the programming of organ morphogenesis and downy mildew resistance. CsIVP directly targets well-known developmental regulators to mediate vascular configuration and organ development. On the other hand, CsIVP negatively regulates SA production and works together with CsNIMIN1 to repress *PR-1* gene expression, compromising pathogen resistance in cucumber.

## Results

### Ancient origin of CsIVP and its expression in vascular tissues

**Fig 1:**
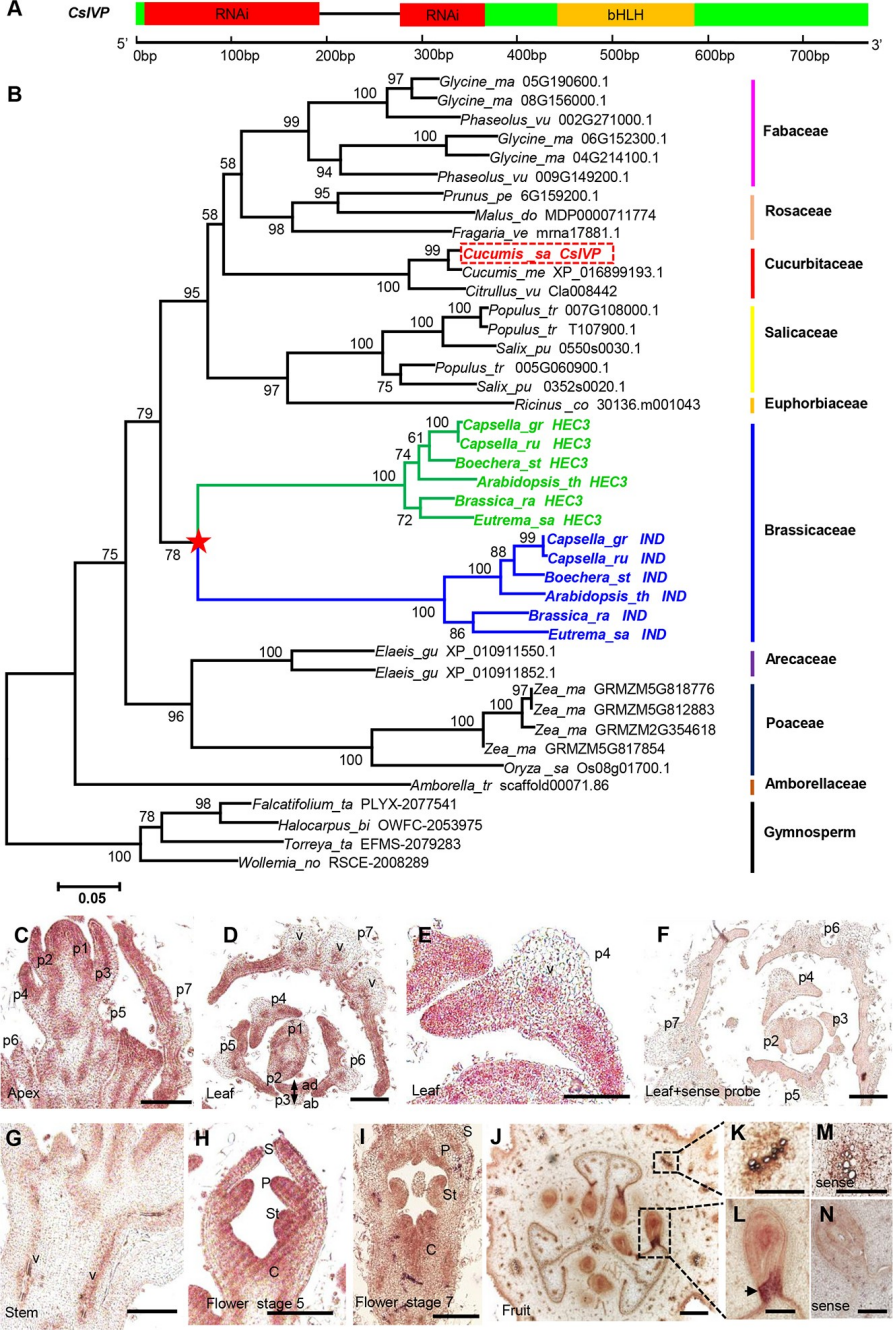
Gene structure, phylogenetic tree, and expression analysis of *CsIVP*. (A) *CsIVP* gene structure in which exons and the intron are indicated by green boxes and a black line, respectively; red box indicates the RNAi-targeted region, and the orange box represents the bHLH domain. (B) Phylogenetic analysis of *CsIVP* and its homologs. *CsIVP* is highlighted in red, and the star indicates the duplication event of *HEC3* in the Brassicaceae family. Gymnosperm sequences were used as the outgroup. Bootstrap values over 50% are placed above the branches. (C–N) RNA in situ hybridization analysis of *CsIVP* in cucumber. (C–F) *CsIVP* transcripts are highly expressed in the shoot apical meristem and leaf primordia. (C) Longitudinal section of a cucumber shoot apex, (D) transverse section of a shoot apex, (E) enlarged view of the leaf primordia p4 in panel D, (F) transverse section of a shoot apex hybridized with the *CsIVP* sense probe. p1–p7: leaf primordia 1–7. (G–I) *CsIVP* is expressed in stem vascular strands (panel G) and floral organ primordium (panels H–I). (J–N) *CsIVP* transcripts are accumulated in fruit vascular tissues and at the boundary between the developing seed and the fruit placenta (arrow in panel L) (panels K–L). (M, N) In situ hybridization with the *CsIVP* sense probe. Scale bars represent 200 μm in panels C–D and F–I, 100 μm in panel E, 50 μm in panels J–K and M, and 25 μm in panels L and N. bHLH, basic Helix-Loop-Helix; C, carpel; *CsIVP*, *Cucumis sativus Irregular Vasculature Patterning*; *HEC3*, *HECATE3*; P, petal; RNAi, RNA interference; S, sepal; St, stamen; v, vascular tissue.

Previously, we performed laser microdissection-transcriptome profiling to identify candidate genes regulating phloem development in cucumber. Among them, we discovered a putative basic Helix-Loop-Helix (bHLH) transcription factor—Csa6G483450—that encodes 224 amino acid residues and exhibited high expression in the fruit [27] (Fig 1A). Based on its vasculature patterning phenotype in RNA interference (RNAi) lines and phylogeny, we named this transcription factor, *C**ucumis* *s**ativus* *I**rregular* *V**asculature* *P**atterning* (*CsIVP* hereafter). *CsIVP* belongs to the *HECATE3* (*HEC3*) subfamily, which together with the *HEC1/2* genes were generated by a *HEC* duplication prior to the origin of angiosperms; these genes were present in early mosses, lycophytes, and gymnosperms (S1A Fig). *HEC3* underwent further expansion in certain angiosperm families (Fig 1B). A gene duplication event, in the Brassicaceae family, gave rise to *HEC3* and *INDEHISCENT* (*IND*) (Fig 1B), with HEC3 (46.4% identical to CsIVP) functioning in stigma and transmitting tract development and IND (37.2% identical to CsIVP) regulating valve margin identity and fruit opening in *Arabidopsis* [28,29].

*CsIVP* is expressed in young leaf and stem tissue (S1B and S1C Fig) and has a high expression level in young fruits (S1D Fig). Among the 4 fruit tissues (exocarp, mesocarp, endocarp, and ventricle), *CsIVP* transcript levels increased from exocarp to ventricle (S1E Fig). In situ hybridization assays showed that *CsIVP* mRNA accumulated in the shoot apical meristem, leaf primordia, young stem, floral organs, and the boundary between the developing seed and the placenta, especially in vascular tissues of those organs (Fig 1C–1N). As expected, green fluorescent protein (GFP)-tagged CsIVP was localized to the nucleus (S1F Fig).

### *CsIVP* regulates cucumber organ morphogenesis and reproduction

**Fig 2:**
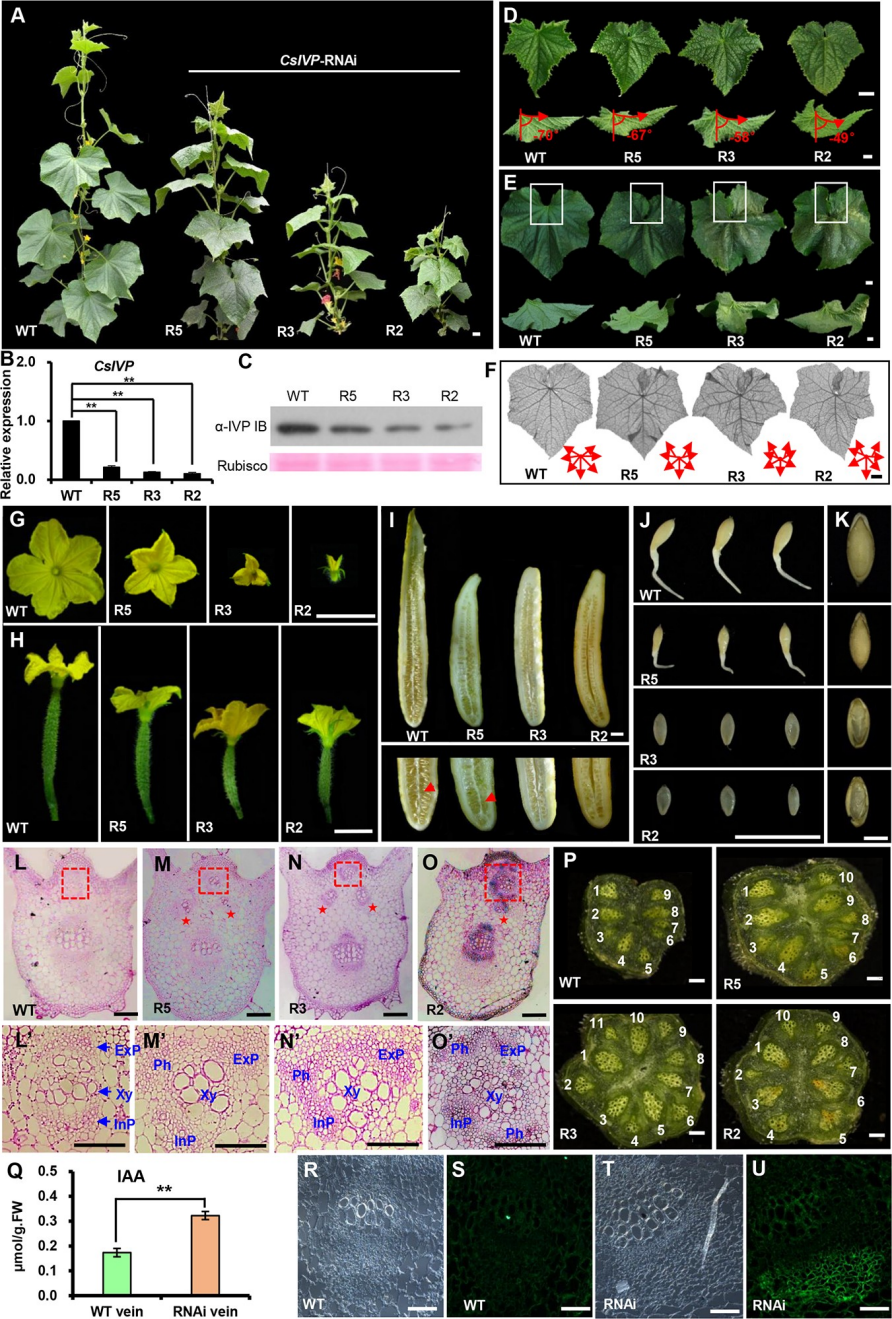
Phenotypic characterization of *CsIVP*-RNAi transgenic plants. (A) Plant morphology of WT and *CsIVP-*RNAi lines R5, R3, and R2. (B) qRT-PCR analysis indicates reduced expression of *CsIVP* in the *CsIVP*-RNAi lines. (C) Immunoblot blot analysis indicates reduced CsIVP protein in the *CsIVP*-RNAi lines. (D–E) Morphology of young (panel D) and mature leaves (panel E) of WT and *CsIVP*-RNAi lines. White squares represent the gap between the bilateral leaf margins. The angle between the vertical axis and the primary vein indicates the degree of down-curled leaf. (F) Leaf venation in WT and *CsIVP*-RNAi leaves. Red arrows represent the primary veins in the leaf. (G) Male flower size at anthesis. (H) Fruit at anthesis in WT and *CsIVP*-RNAi lines. (I) Reduced mature fruit length and decreased seed viability in the *CsIVP*-RNAi lines. (J) Seeds after 36 h of germination. (K) Seeds after testa removal. (L–O) Transverse sections of leaf mid-veins of WT (panel L) and *CsIVP*-RNAi (panels M–O) plants. Red stars in panels M–O indicate extra vascular bundles in *CsIVP*-RNAi lines. (L’–O’) Amplified vascular bundles in red boxes of panels L–O. (P) Transverse sections of stems from WT and *CsIVP*-RNAi lines. White numbers indicate the vascular bundles. (Q) IAA content in leaf veins of WT and *CsIVP*-RNAi transgenic plants. (R–U) IAA distribution in leaf veins as detected by immunolocalization. Anti-IAA monoclonal antibodies and DyLight 488–conjugated goat anti-mouse IgG antibodies were used to detect IAA. (R, T) Differential interference contrast images; (S, U) fluorescent images. Scale bars represent 2 cm in panels A, D–J, and P; 2 mm in panel K; 200 μm in panels L–O; 50 μm in panels L’–O’; and 100 μm in panels R–U. Values are means ± SE (*n* = 3) in panels B and Q. Double asterisks indicate significant difference at *P* < 0.01 by *t* test. The data underlying this figure are included in S1 Data. *CsIVP*, *Cucumis sativus Irregular Vasculature Patterning*; ExP, external phloem; IAA, indole-3-acetic acid; IgG, immunoglobulin G; InP, internal phloem; Ph, phloem; qRT-PCR, quantitative real-time PCR; RNAi, RNA interference; WT, wild type; Xy, xylem.

To gain insight into *CsIVP* functions in cucumber, an RNAi construct—specifically targeting a 270-bp sequence unique to *CsIVP*—was generated (Fig 1A) and delivered into the cucumber inbred line, R1461, via *Agrobacterium tumefaciens–*mediated cotyledon transformation [30]. Due to unexpectedly high lethality, a total of 7 independent transgenic lines were recovered and self-crossed for another 2 generations for seed propagation and phenotypic characterization; 3 representative lines (R5 mild, R3 medium, and R2 severe) were selected for further study (Fig 2A). Gene expression analysis indicated efficient knockdown of the *CsIVP* transcripts, with 79%, 87%, and 90% reduction observed in R5, R3, and R2, respectively (Fig 2B). In addition, immunoblot analyses showed that CsIVP protein accumulated to a lower level in *CsIVP*-RNAi transgenic lines compared to wild-type (WT) (Fig 2C). Importantly, no significant differences were detected in the expression of related *CsHEC1* and *CsHEC2* genes between the *CsIVP*-RNAi transgenic lines and WT plants (S2A and S2B Fig).

In comparison to WT, *CsIVP-*RNAi plants were dwarf with fasciated stems (Fig 2A, S2C and S2D Fig), and their internode lengths were greatly reduced, resulting in a 28% to 76% decrease in plant height (S2E Fig); conversely, the stem diameter was increased by 20% to 29% (S2F Fig). Leaves of these *CsIVP*-RNAi plants were downwardly curled (Fig 2A, 2D and 2E). At the junction between the petiole and midvein of WT young leaves, the angle between the vertical axis and the midvein was approximately −70.4 ± 1.1° (*n* = 6), whereas it was only −48.9 ± 2.4° (*n* = 6) in the severe transgenic line R2 (Fig 2D). In WT leaves, the midvein extended out in an almost straight line, whereas in the transgenic leaves, the midvein was curved, becoming more severe in older leaves (Fig 2D and 2E). Furthermore, the WT leaves had a gap between the bilateral leaf margins (Fig 2E, white box in WT). However, in the *CsIVP*-RNAi transgenic leaves, these leaf margins overlapped, resulting in no visible gap (Fig 2E, white boxes in R5, R3, R2). Next, we cleared leaves to view leaf venation patterns, and here, we ascertained that the angles between primary veins were enlarged, and more secondary veins were formed in *CsIVP*-RNAi leaves, compared to WT (Fig 2F).

During reproductive growth, male flowers were much smaller and—during anthesis—were mostly partially open or even closed in the R3 and R2 lines (Fig 2G and S2G Fig). In addition, stamens barely released pollen (S2K and S2K’ Fig), and pollen viability decreased from 95.3% in WT (*n* = 300) to 86.5% (*n* = 223), 28.5% (*n* = 298), and 5.6% (*n* = 213) in R5, R3, and R2, respectively (S2L Fig). Similarly, at anthesis, ovary length of transgenic female flowers was significantly reduced, while ovary diameter remained unchanged (Fig 2H, S2H and S2I Fig). After self-pollination, the mature fruit was shorter and produced significantly fewer seeds in the 3 *CsIVP*-RNAi transgenic lines (red arrows in Fig 2I and S2J Fig). In contrast to WT, seeds were smaller in line R5 and shrunken in lines R3 and R2 plants (S2M Fig). Seed germination tests showed that the WT root reached approximately 3.1 ± 0.23 cm (*n* = 10) in length, after 36-h incubation at 28°C, whereas the R5 root was much shorter, approximately 1.1 ± 0.17 cm (*n* = 10), and R3 and R2 seeds remained in the process of imbibition with an absence of root protrusion (Fig 2J). The germination rates for the R5, R3, and R2 lines were 95% (*n* = 20), 35% (*n* = 20), and 16% (*n* = 12), respectively; these low rates were consistent with the fact that, for the R3 and R2 seeds, 60% (*n* = 35) and 83% (*n* = 30), respectively, were embryo free (Fig 2K). These data indicated that knockdown of *CsIVP* led to developmental defects, including both organ morphogenesis and reproduction.

### Knockdown of *CsIVP* perturbed vascular development and increased auxin accumulation in leaf veins

Given that *CsIVP* expression was enriched in vascular tissues (Fig 1C–1N), we further examined the vascular architecture. The WT midvein consists of 2 fascicular vascular bundles; within each vascular bundle, the external phloem, central xylem, and inner phloem forms a typical bicollateral structure (Fig 2L and 2L’), whereas the leaf midveins of *CsIVP*-RNAi plants had one or two additional vascular bundles (red stars in Fig 2M, 2N and 2O). The extra vasculature phenotype was observed in 85%, 70%, and 65% mature leaves in R2, R3, and R5 lines, respectively. Furthermore, in these *CsIVP*-RNAi plants, the bicollateral structure was disorganized, with improper position of xylem and phloem, and more phloem cells surrounding the central xylem (Fig 2M’, 2N’ and 2O’). Vascular defects were observed also in the stem (Fig 2P). Compared to 9 vascular bundles in the WT stem, the *CsIVP*-RNAi stem contained 10 to 11 vascular bundles, with each being enlarged in size (Fig 2P). In the transgenic fruit, the size of each vascular bundle was decreased, although the number of vascular bundles was unchanged (S2N and S2O Fig). These findings implicated CsIVP as an important regulator for vascular architecture in cucumber.

Given that hormones are critical signaling components that can circulate within the vascular system, and are involved in vascular development [31,32], we next measured the levels of endogenous auxin (indole-3-acetic acid [IAA]), cytokinin (zeatin riboside [ZR]), gibberellic acid3 (GA3), brassinosteroids (BRs), and abscisic acid (ABA) in the veins of WT and *CsIVP*-RNAi leaves. Although no differences were observed in the levels of ZR, GA3, BR, and ABA between WT and transgenic plants (S2P Fig), we detected increased levels of IAA in leaf veins of *CsIVP-*RNAi plants (Fig 2Q). At the cellular level, we detected stronger IAA signals in the phloem and xylem tissues of *CsIVP-*RNAi leaf veins compared with WT (Fig 2R, 2S, 2T and 2U). Equivalent IAA levels were detected in fruits of WT and *CsIVP-*RNAi plants (S2Q, S2R, S2S and S2T Fig).

### CsIVP directly promotes the expression of vascular-related genes

**Fig 3:**
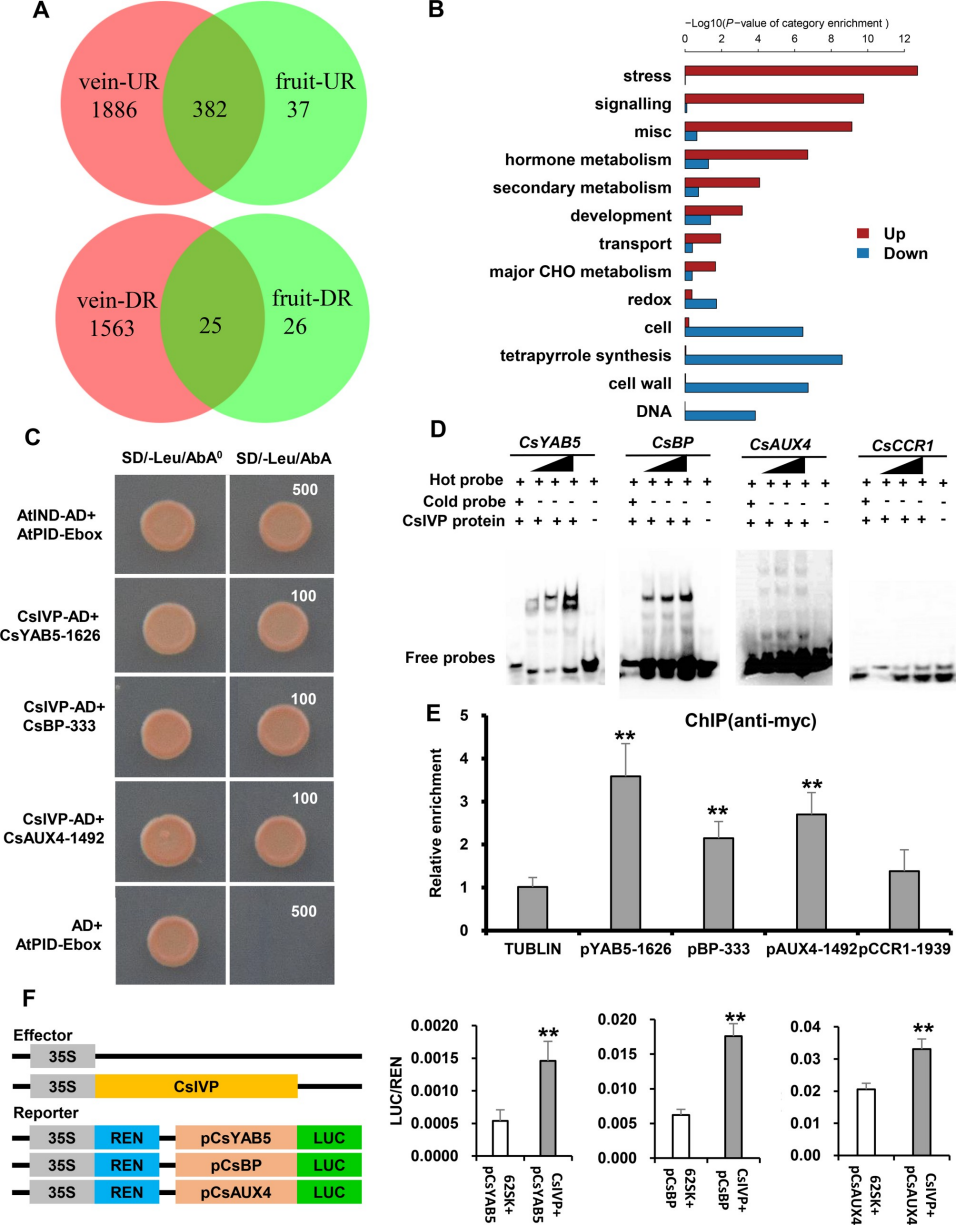
Transcriptome and interaction analysis between CsIVP and putative downstream vascular-related targets. (A) Venn diagrams of the overlapping DEGs that were up-regulated or down regulated in the vein and fruit of *CsIVP*-RNAi plants, as compared to WT plants. (B) Gene category enrichment of up- and down-regulated genes in the vein of *CsIVP* transgenic compared to WT plants. (C) Yeast one-hybrid assays identify interactions between CsIVP and the E-box from the *CsYAB5*, *CsBP*, and *CsAUX4* promoters. Activation of AbAr occurred when CsIVP bound to the E-box sequence. The SD/-Leu medium with 100 ng/ml or 500 ng/ml inhibitory AbA was used to screen for interactions. (D) Visualization of direct binding of CsIVP to promoters of *CsYAB5*, *CsBP*, *CsAUX4*, and *CsCCR1* via EMSAs. Three concentrations of labeled probe were used (80, 120, and 160 fmol). Cold (unlabeled) probes were used as competitors. (E) ChIP-PCR showing the in vivo binding of CsIVP to the pCsYAB-1626, pCsBP-333, and pCsAUX4-1492 promoters. The cucumber alpha-tubulin gene (*CsTUBULIN*, GenBank: AJ715498) was used as the internal gene control, the pCsCCR1-1939 was used as a negative amplification. Values are means ± SE (*n* = 3). Double asterisks indicate significant difference at *P* < 0.01 by *t* test. (F) Luciferase activity measured in tobacco leaves after co-expression of *35S*:*CsIVP* with *proCsYAB5*:*LUC*, or *proCsBP*:*LUC*, or *proCsAUX4*:*LUC*. Values are means ± SE (*n* = 6). Double asterisks indicate significant difference at *P* < 0.01 by *t* test. The data underlying this figure are included in S2 Data. AbA, aureobasidin A; AbAr, AbA resistance gene; AUX4, AUXIN/INDOLEACETIC ACIDS4; BP, BREVIPEDICELLUS; CCR1, CINNAMOYL COA REDUCTASE1; ChIP, chromatin immunoprecipitation; *Cs*, *Cucumis sativus*; *CsIVP*, *Cucumis sativus Irregular Vasculature Patterning*; DR, down-regulated; EMSA, electrophoretic mobility-shift assay; RNAi, RNA interference; SD/-Leu, synthetical dropout/-leucine; UR, up-regulated; WT, wild type; *YAB5*, *YABBY5*.

To explore the regulatory networks by which *CsIVP* mediates in vascular development, RNA sequencing (RNA-seq) was performed for leaf veins and fruit (4 d before anthesis) from WT and R5 transgenic plants. Here, 21.58 to 27.13 million paired-end reads were generated for each sample (S1 Table). Analysis of these datasets showed that—relative to WT—2,268 and 1,588 genes were up- and down-regulated, respectively, in the leaf vein of *CsIVP* transgenic plants (Fig 3A and S2 Table). Functional category enrichment analysis indicated that genes related to stress, signaling, hormone metabolism, and development were significantly enriched among the 2,268 up-regulated genes in the leaf vein of *CsIVP*-RNAi (Fig 3B). As expected, expression of many well-known developmental regulators, and auxin-related genes, were significantly altered in the veins of *CsIVP*-RNAi compared with WT plants, including *YAB5*, *BP*, *KNAT2*, *KNAT6*, *FIL* (*FILAMENTOUS FLOWER*), *APL*, *IRX6*, and *AUXIN/INDOLEACETIC ACIDS4* (*AUX4*) (S3 and S4 Tables). Our quantitative real-time PCR (qRT-PCR) assays confirmed the differential expression of 6 vascular-related genes (S3A Fig), suggesting that *CsIVP* may regulate organ development through interactions with other developmental regulators.

Based on our RNA-seq data—and the presence of the E-box *cis*-element (CANNTG) previously shown to act as the binding site for bHLH transcription factors [33]—11 cucumber genes were chosen for yeast one-hybrid assays (S3B Fig and S5 Table). As shown in Fig 3C, CsIVP bound to the E-box in the promoters of vascular-related genes, including *C*. *sativus* (*Cs*)*YAB5* (-1626, CAATTG), *CsBP* (-333, CAACTG), *CsAUX4* (-1492, CACATG), and *CINNAMOYL COA REDUCTASE1* (*CsCCR1*) (-1939, CACTTG). These binding activities were validated by electrophoretic mobility-shift assays (EMSAs), using a 30- to 40-bp region containing the E-box, as probe. Although no interaction with *CsCCR1* was observed, CsIVP bound strongly to *CsBP* and moderately to *CsAUX4* and *CsYAB5* (Fig 3D). We also performed chromatin immunoprecipitation (ChIP)-PCR assays to confirm the bindings, in vivo, using the CsIVP-c-myc (MYC) transgenic cucumber. Our results showed that CsIVP substantially enhanced the real-time PCR-based detection of the *CsBP*, *CsAUX4*, and *CsYAB5* promoters after immunoprecipitation (Fig 3E). A transactivation assay was also performed to further validate these interactions, using the luciferase/renilla (LUC/REN) system. When expressed in *Nicotiana benthamiana* leaves, CsIVP acted as a positive regulator and enhanced the expression of *pCsYAB5*::LUC, *pCsBP*::LUC, and *pCsAUX4*::LUC (Fig 3F), consistent with the down-regulation of *CsBP*, *CsAUX4*, and *CsYAB5* in the *CsIVP-*RNAi plants (S3A Fig). These findings support the hypothesis that CsIVP directly binds to the promoters of *CsYAB5*, *CsBP*, and *CsAUX4* and promotes their expression. Importantly, no interactions were detected between related CsHEC1 or CsHEC2 protein and the promoters of *CsYAB5*, *CsBP*, and *CsAUX4*, based on our yeast one-hybrid and LUC/REN transactivation assays (S3C, S3D, S3E and S3F Fig).

### Knockdown of *CsYAB5* mimics the *CsIVP*-RNAi phenotype in cucumber

**Fig 4:**
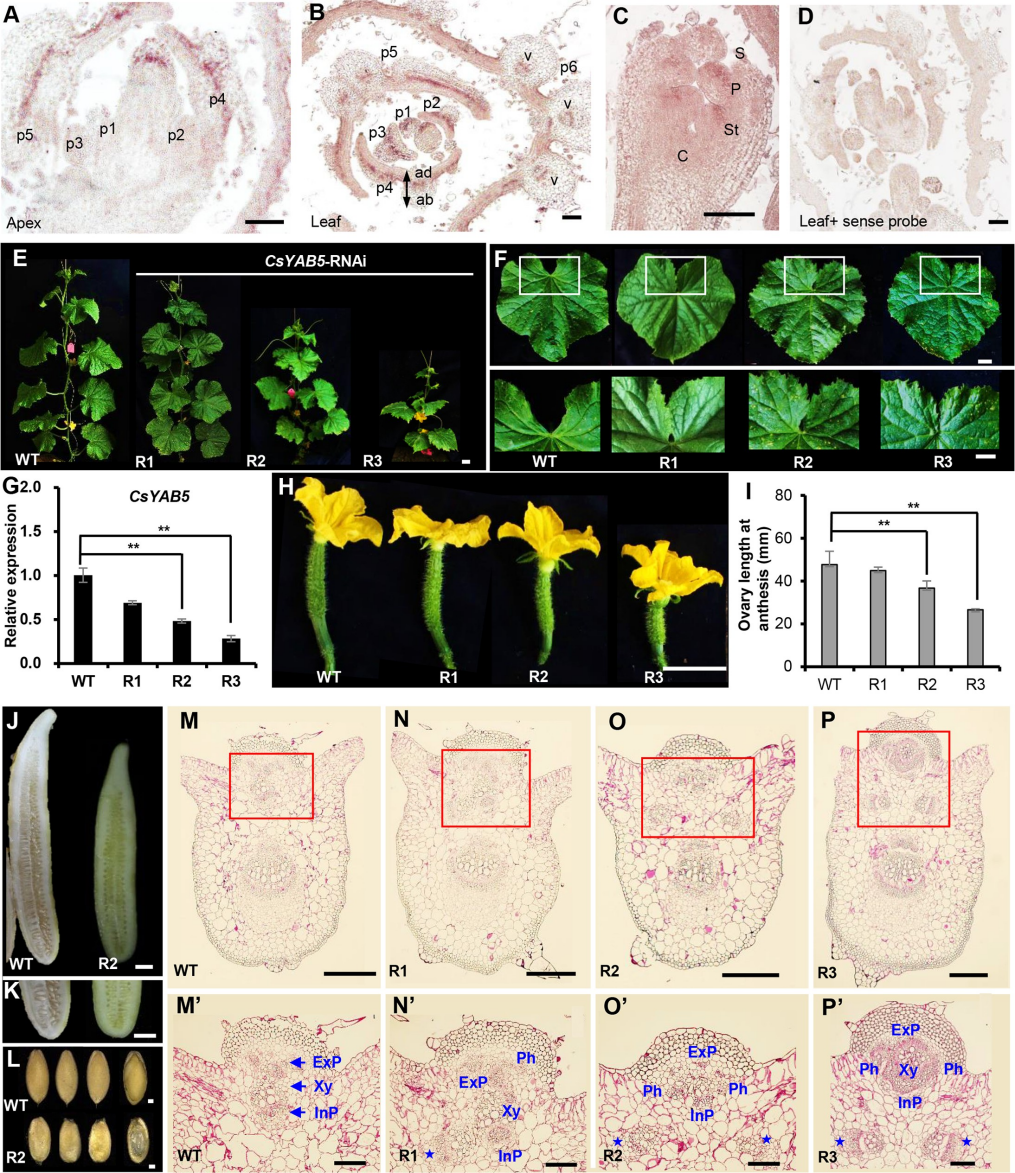
Functional characterization of *CsYAB5* in cucumber. (A–D) RNA in situ hybridization analysis of *CsYAB5* in the cucumber shoot apex and flower buds. Longitudinal (panel A) and transverse sections (panel B) of cucumber shoot apex. p1–p6: leaf primordia 1–6. (C) Longitudinal image of a flower bud. (D) Transverse section of leaf primordia in the shoot apex, hybridized with the *CsYAB5* sense probe. (E) Plant morphology of WT and *CsYAB5*-RNAi line R1, R2, and R3. (F) Leaf morphology of WT and *CsYAB5*-RNAi lines. White squares show the gap between the bilateral leaf margins. (G) qRT-PCR analysis indicates reduced expression of *CsYAB5* in the *CsYAB5*-RNAi lines. (H–I) Fruit at anthesis in WT and *CsYAB5*-RNAi lines (panel H); quantification of ovary length at anthesis (panel I). (J–K) Reduced mature fruit length (panel J) and decreased seed viability (panel K) in the *CsYAB5*-RNAi line. (L) Morphology of mature seeds of WT and *CsYAB5*-RNAi line. (M–P) Transverse sections of leaf mid-veins of WT (panel M) and *CsYAB5*-RNAi (panels N–P) plants. (M’–P’) Amplified vascular bundles in red boxes of panels M–P. Blue stars in panels N’–P’ indicate extra vascular bundles in *CsYAB5*-RNAi lines. Scale bars represent 100 μm in panels A–D; 2 cm in panels E–F, H, and J–K; 1 mm in panel L; 500 μm in panels M–P; and 200 μm in panels M’–P’. Values are means ± SE (*n* = 3) in panels G and I. Double asterisks indicate significant difference at *P* < 0.01 by *t* test. The data underlying this figure are included in S3 Data. C, carpel; *Cs*, *Cucumis sativus*; ExP, external phloem; InP, internal phloem; P, petal; Ph, phloem; qRT-PCR, quantitative real-time PCR; RNAi, RNA interference; S, sepal; St, stamen; v, vascular tissue; WT, wild type; Xy, xylem; *YAB5*, *YABBY5*.

To further address the regulatory network of *CsIVP* in vascular development, its direct downstream target gene, *CsYAB5*, was selected for further characterization in cucumber. Similar to *CsIVP*, *CsYAB5* is expressed in the young leaf, stem, and fruit (S4A, S4B and S4C Fig). In situ hybridization assays showed that *CsYAB5* signal is enriched in the abaxial side of the leaf, petal, and stamen primordia, as well as in the vascular tissues of the leaf vein (Fig 4A, 4B and 4C). Such an expression pattern is similar to the *CsYAB5* counterpart in *Arabidopsis* and maize [34,35]. No signal was detected when hybridization with the sense probe of *CsYAB5* (Fig 4D). Moreover, we constructed *CsYAB5*-RNAi transgenic plants and characterized their developmental phenotypes. Compared to WT plants, *CsYAB5*-RNAi plants exhibited reduced height (Fig 4E) and abnormal leaf morphology with no visible gap between the bilateral leaf margins (Fig 4F). Expression analyses indicated that *CsYAB5* was reduced by 31% to 72% in the *CsYAB5*-RNAi lines (Fig 4G). The fruit length was significantly shorter, and the degree of fruit length reduction was consistent with the decrease in *CsYAB5* expression (Fig 4H, 4I and 4J). The seeds were also smaller and shrunken in *CsYAB5*-RNAi plants (Fig 4K and 4L). Notably, transverse sectioning of leaf veins of *CsYAB5*-RNAi plants showed similar phenotypes to *CsIVP*-RNAi plants (Fig 4M, 4N, 4O and 4P), including 1 or 2 additional vascular bundles (blue stars in Fig 4M’, 4N’, 4O’ and 4P’) and improper positioning of xylem and phloem (Fig 4N’, 4O’ and 4P’). These results, together with the interaction data, suggested that CsYAB5 functions downstream of CsIVP to regulate vascular patterning and organ morphogenesis in cucumber.

### *CsIVP*-RNAi plants are resistant to downy mildew

**Fig 5:**
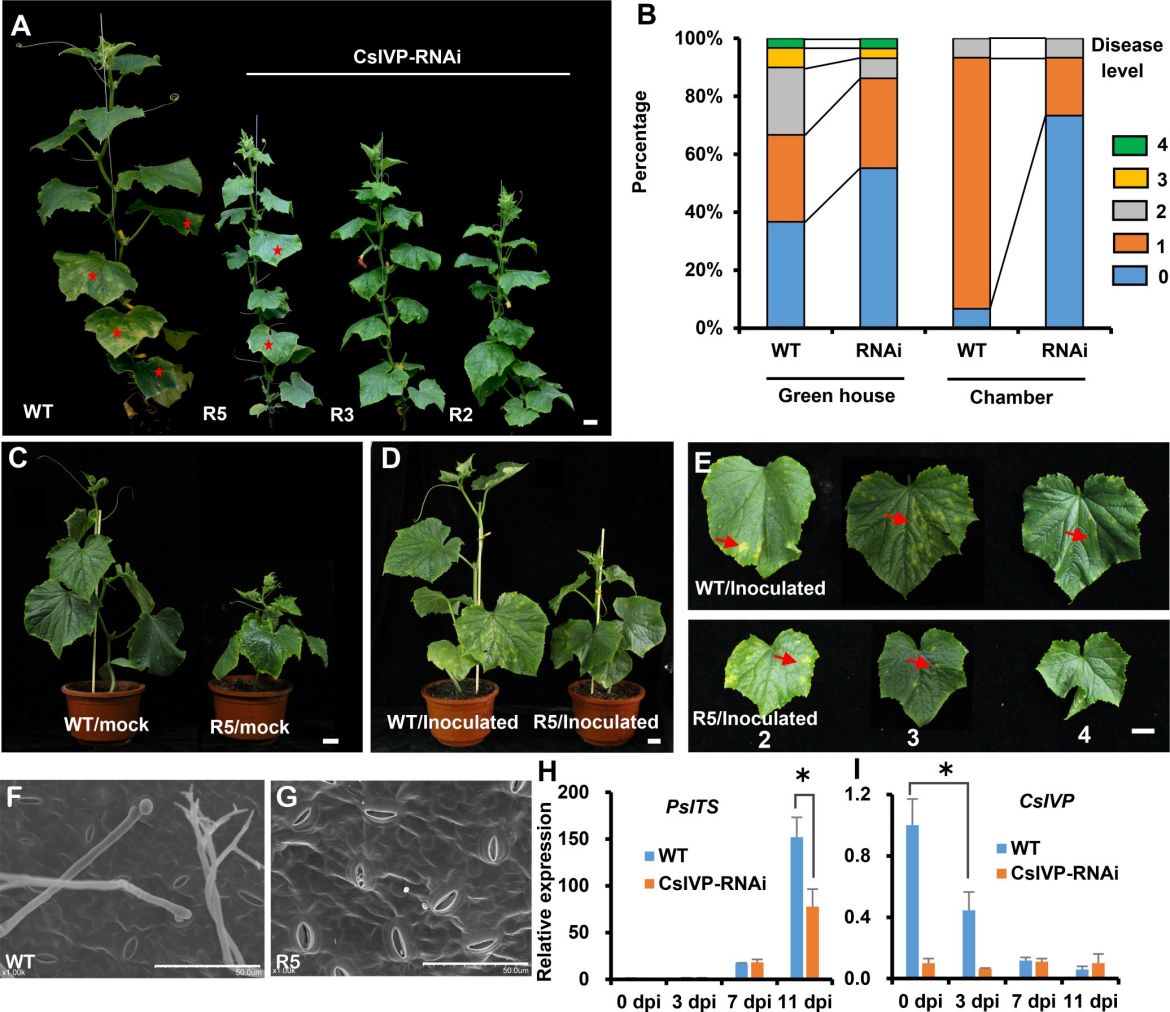
Response of *CsIVP*-RNAi plants to pathogen infection. (A) Increased tolerance of *CsIVP*-RNAi plants to pathogen attack during growth under greenhouse conditions. (B) Disease level of WT and *CsIVP*-RNAi plants under greenhouse and growth chamber conditions. (C–E) Phenotypic characterization of WT and *CsIVP*-RNAi plants 11 dpi with *Pseudoperonospora cubensis* in a growth chamber. The second, third, and fourth leaves (from bottom to top) are displayed in panel E. (F–G) Sporulation of *P*. *cubensis* on WT and *CsIVP*-RNAi plants imaged by scanning electron microscopy. (H) *PsITS* expression after inoculation with *P*. *cubensis* at 0, 3, 7, and 11 dpi. (I) *CsIVP* expression after inoculation with *P*. *cubensis* at 0, 3, 7, and 11 dpi. Scale bars represented 2 cm in panels A and C–E and 50 μm in panels F–G. Values are means ± SE (*n* = 3) in panels H–I. Asterisk indicate significant difference at *P* < 0.05 by *t* test. The data underlying this figure are included in S4 Data. *CsIVP*, *Cucumis sativus Irregular Vasculature Patterning*; dpi, days post inoculation; *PsITS*, *P*. *cubensis internal transcribed spacer*; RNAi, RNA interference; WT, wild type.

Cucumber plants are vulnerable to various pathogens, especially during late-season production; downy mildew is one of the most devastating diseases in cucumber production [36]. During seed propagation, we noticed that plants of all 3 *CsIVP*-RNAi lines (R2, R3, and R5) were exceptionally resistant to greenhouse diseases (Fig 5A). WT plants showed severe symptoms of downy mildew, with angular chlorotic lesions throughout the foliage (Fig 5A, red stars). In contrast, symptoms in *CsIVP*-RNAi R5 plants were greatly reduced, and R3 and R2 lines only showed mild symptoms (Fig 5A, red star in R5). The field disease index of *CsIVP*-RNAi plants is lower than WT (Fig 5B and S5A Fig).

Due to extremely low germination rates for the R3 and R2 lines, the R5 line was selected for further study. To investigate the roles of CsIVP on disease resistance, 7 leaf-stage seedlings were grown in a controlled environment chamber and inoculated with *P*. *cubensis*, a type of water mold that causes downy mildew in cucumber [36]. Consistently, *CsIVP*-RNAi plants were more resistant to *P*. *cubensis* (Fig 5C, 5D and 5E). Eleven days after inoculation, the second, third, and fourth true leaves of WT plants displayed obvious angular chlorotic lesions, whereas only moderate lesions occurred on the second and third true leaves of *CsIVP*-RNAi plants (Fig 5C, 5D and 5E). Some 73% (*n* = 15) of leaves exhibited no disease in *CsIVP*-RNAi plants, whereas only 7% (*n* = 15) of leaves showed no visible disease in WT (Fig 5B). The disease index was 11.1 for WT and 3.7 for *CsIVP*-RNAi plants (S5A Fig). Moreover, by scanning electron microscopy, visible conidiophores were observed in WT, but not in *CsIVP*-RNAi plants (Fig 5F and 5G). Consistently, the level of the *P*. *cubensis internal transcribed spacer* (*PsITS*) gene [37], which is used to assess pathogen growth on the host plants, was increased 11 days post inoculation (dpi) in WT but was significantly lower in *CsIVP*-RNAi plants (Fig 5H). In addition, at 3 dpi, the *CsIVP* transcript level was decreased greatly in WT and was reduced to the level in *CsIVP*-RNAi plants after 7 dpi (Fig 5I). These data suggested that CsIVP is involved in downy mildew resistance in cucumber.

### *CsIVP* regulates pathogen resistance though interacting with CsNIMIN1

**Fig 6:**
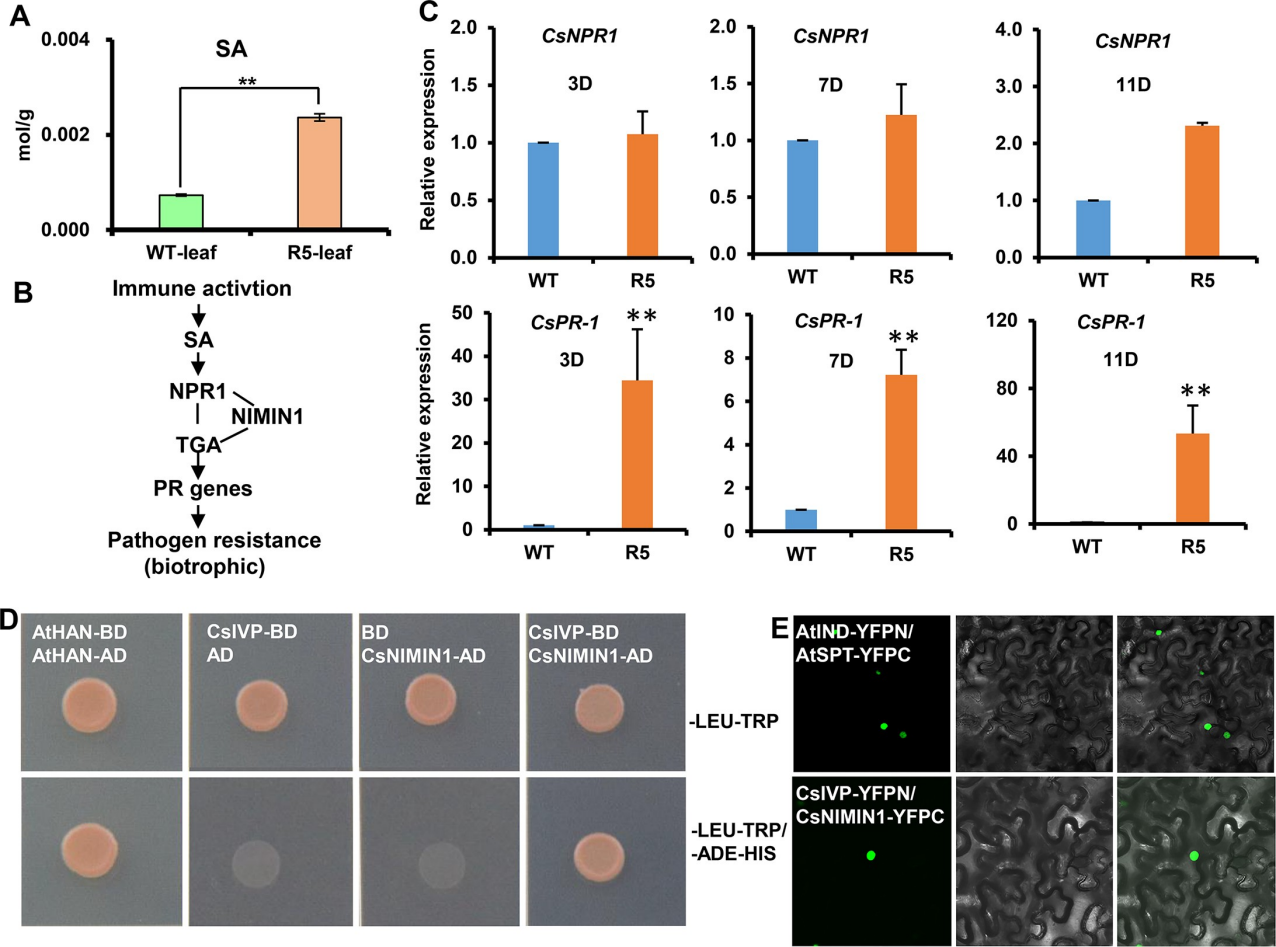
Interaction of CsIVP with CsNIMIN1, a repressor in the SA-responsive pathway. (A) SA content in leaf veins of WT and *CsIVP*-RNAi transgenic plants. (B) Schematic diagram of the major components in the SA-mediated plant defense pathway. (C) Expression analyses of selected genes involved in the induced systemic responses in WT and *CsIVP*-RNAi plants, at the times indicated after *P*. *cubensis* inoculation. (D–E) Physical interactions between CsIVP and CsNIMIN1, as revealed by Y2H (panel D) and BiFC assays (panel E). Protein interactions are indicated by green YFP fluorescent signals in nuclei (left panels); differential interference contrast images of tobacco cells are shown in the middle panels; and merged channels are shown in right panels. Values are means ± SE (*n* = 3) in panels A and C. Double asterisks indicate significant difference at *P* < 0.01 by *t* test. The data underlying this figure are included in S5 Data. BiFC, bimolecular fluorescence complementation; *Cs*, *Cucumis sativus*; *CsIVP*, *Cucumis sativus Irregular Vasculature Patterning*; NIMIN1, NIM1-INTERACTING 1; RNAi, RNA interference; SA, salicylic acid; WT, wild type; Y2H, yeast two-hybrid; YFP, yellow fluorescent protein.

To further study how *CsIVP* mediates in pathogen resistance, the differentially expressed genes (DEGs) in WT and *CsIVP*-RNAi plants were classified by MapMan (S5B Fig). Interestingly, genes involved in the SA, ethylene, jasmonic acid (JA), and ABA signaling pathways were significantly up-regulated in *CsIVP*-RNAi plants, providing evidence for a putative molecular mechanism of disease resistance in *CsIVP*-RNAi plants (S5B Fig). In addition, we observed a particular enrichment of defense-related genes in the *CsIVP*-RNAi line in our transcriptome data, as indicated by the significantly enriched gene ontology (GO) terms (S5C Fig). As expected, the biotrophic pathogen resistance hormone SA was significantly increased in *CsIVP*-RNAi leaves (Fig 6A). The expression of *PR-1* in the SA signaling pathway was also significantly increased in *CsIVP*-RNAi plants after inoculation with *P*. *cubensis* (Fig 6B and 6C). These data support the hypothesis that, in cucumber, *CsIVP* regulates pathogen resistance, via the SA signaling pathway. We next screened 8 proteins as potential CsIVP partners, using the yeast two-hybrid (Y2H) assay (S6A Fig). CsIVP exhibited strong interactions with CsNIMIN1, a repressor in the SA signaling pathway (Fig 6D) [25], and weak interactions were observed with *C*. *sativus* WHIRLY 1 (CsWHY1) and SUPPRESSOR OF NPR1 (CsSNI1) (S6A Fig) [24], but no interactions were detected with the remaining 5 tested proteins (S6A Fig).

To confirm these protein interactions, in vivo, we next performed bimolecular fluorescence complementation (BiFC) assays in *N*. *benthamiana* leaves. A positive interaction was detected between CsIVP and CsNIMIN1 (Fig 6E), but no interaction was observed with CsWHY1 or CsSNI1. To further confirm that these interactions were specific to CsIVP, another 2 HEC family proteins, CsHEC1 and CsHEC2, were examined. An interaction between CsHEC1/2 with CsNIMIN1 was not detected in both Y2H and BiFC assays (S6B and S6C Fig). Taken together, these data support the hypothesis that CsIVP participates in downy mildew resistance through the CsNIMIN1-mediated SA response pathways.

## Discussion

Evolution of a vascular system was a primary driving force for land plant diversification and adaptation. The function of the vascular system, in long-distance signaling, is well known. However, the link between vascular development and disease resistance has received much less attention, and few regulators have been identified. In plants, formation of the vascular system underlies organ differentiation and functioning [32]. Therefore, erroneous structuring and configuration of the vascular system, in *CsIVP*-RNAi plants, likely negatively affects a range of developmental processes, thereby causing pleiotropic effects (Fig 2). On a similar note, leaf shape is closely related to the establishment of leaf vasculature [17], and therefore the downward curling of leaves in the *CsIVP*-RNAi plants may reflect the impact of ectopic development of the vasculature (Fig 2). The possibility should also be considered that the broader developmental defects—observed in *CsIVP*-RNAi plants—may reflect a perturbation to vascular development and the interdiction of other, as yet unidentified, *CsIVP* regulatory pathways.

**Fig 7:**
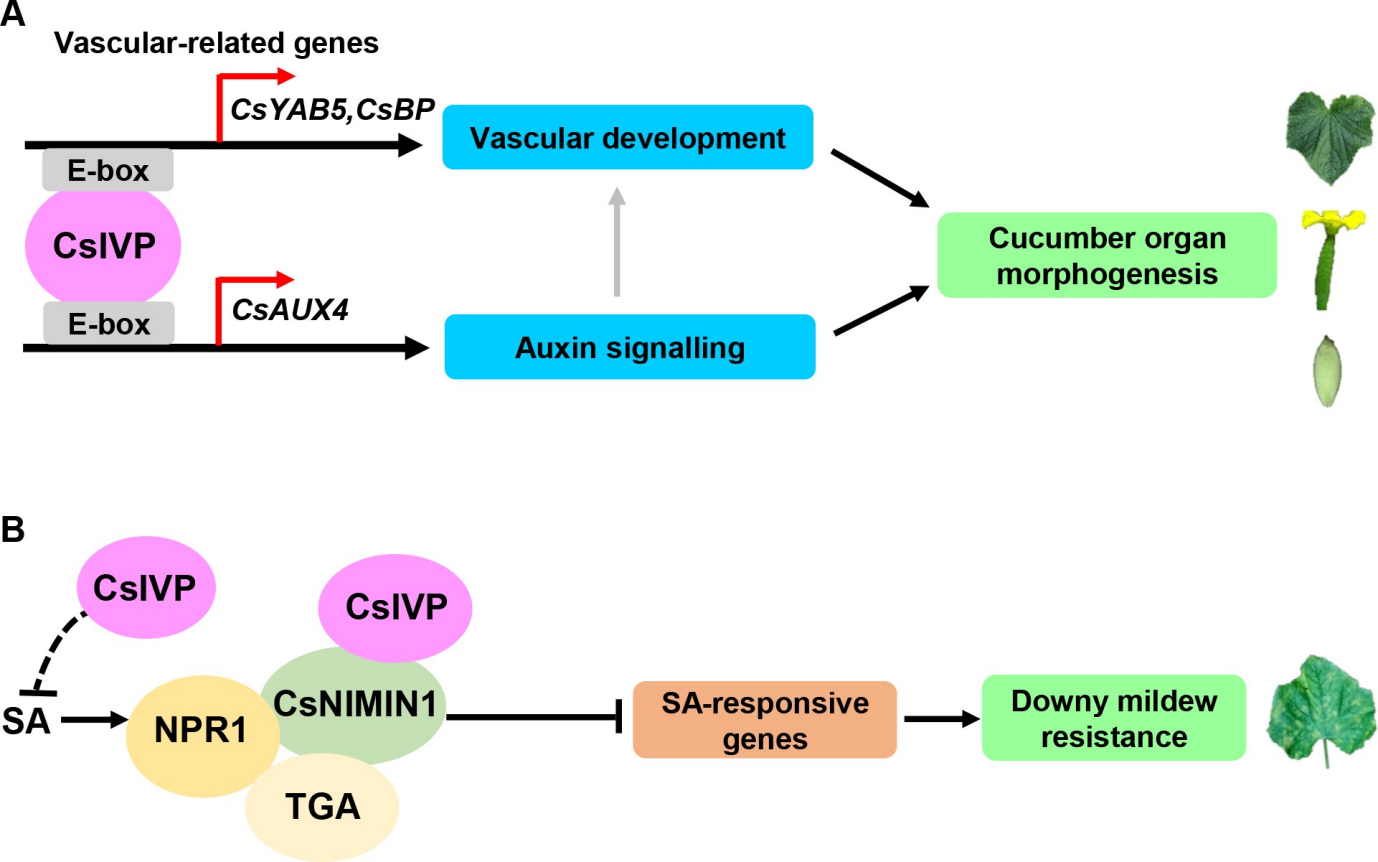
A working model for vasculature regulator *CsIVP* functioning in organ morphogenesis and downy mildew resistance in cucumber. (A) CsIVP regulates organ morphogenesis via 2 pathways, one by directly promoting the expression of vascular-related genes—including *CsYAB5* and *CsBP*—to regulate vascular development, and the other by directly binding to the E-box of *CsAUX4* to mediate auxin signaling. Grey arrow indicates the putative positive role of auxin in vascular development. (B) CsIVP may act as a repressor for SA production and physically interacts with CsNIMIN1 to compromise downy mildew resistance in cucumber. Black arrows, positive regulation; black crosses, inhibition regulation; red arrows, transcription start; black dotted crosses, putative inhibition regulation. *AUX4*, *AUXIN/INDOLEACETIC ACIDS4*; *BP*, *BREVIPEDICELLUS*; *Cs*, *Cucumis sativus*; *CsIVP*, *Cucumis sativus Irregular Vasculature Patterning*; NIMIN1, NIM1-INTERACTING1; SA, salicylic acid; *YAB5*, *YABBY5*.

The CsIVP was shown to directly bind to and promote the expression of *CsYAB5* in cucumber (Fig 3C, 3D and 3F). Previous studies indicated that the plant-specific YAB genes are expressed, abaxially, and play significant roles in leaf development [38,39]. In *Arabidopsis*, mutation of *YAB* genes resulted in loss of abaxial cell identity and narrow leaves [35]. In maize, CRABS CLAW affects leaf length and width, leaf angle, and internode length [34]. In rice, mutation in *Oryza sativa* YABBY4 (OsYAB4) leads to a semi-dwarf phenotype and an abnormal uppermost internode [40]. Here, we established that expression of *CsYAB5*, in the abaxial zone and vascular tissues, resembles *YAB5* in *Arabidopsis* (Fig 4A, 4B and 4C) [41]. Knockdown of *CsYAB5*, by RNAi, led to perturbations in leaf morphology, reduced internodes, and decreased fruit length and fertility, as well as disrupted vascular configuration, which phenocopied *CsIVP*-RNAi plants (Fig 4). These findings support the notion that CsIVP acts as a critical regulator of vasculature patterning and organ morphogenesis, through a direct regulation of *CsYAB5* transcription (Fig 7A).

As leaf serration was observed in *CsYAB5*-RNAi plants (Fig 4), but not in *CsIVP*-RNAi plants (Fig 2); this suggests that *CsYAB5* acts in a distinct pathway, other than through *CsIVP*, in regulating leaf serration. Interestingly, leaf downward curling was obvious in *CsIVP*-RNAi plants (Fig 2) but not in *CsYAB5*-RNAi plants (Fig 4). This downward curling phenotype is similar to the condition observed in *ASYMMETRIC LEAVES2* (*as2*) in *Arabidopsis* (Fig 2A, 2B, 2C, 2D and 2E) [42]. *AS1* and *AS2* form a complex to directly repress the expression of *BP* and *YAB5* in the proximodistal axis of *Arabidopsis* leaves [41]. However, our RNA-seq data indicated no difference in expression of *CsAS1* and *CsAS2* between *CsIVP*-RNAi and WT plants; neither were physical interactions detected between CsIVP and CsAS1 or CsAS2 (S3B Fig). Considering that the *Arabidopsis bp* mutant displays a dwarf plant phenotype and downward-oriented pedicels [43], and CsIVP directly binds to the promoter of *CsBP* and stimulates *CsBP* transcription (Fig 3), CsIVP may function in a novel manner, or in parallel with CsAS1/2, by direct targeting of vascular-related genes—including *CsYAB5* and *CsBP*—during organ morphogenesis in cucumber.

Auxin not only affects organ development but also promotes vascular cell differentiation and vascular strand formation in *Arabidopsis* [31,32]. Hence, the elevated IAA levels detected in the *CsIVP*-RNAi lines may account for their abnormal vascular patterning (Fig 2). Auxin signaling components, AUXIN RESPONSE FACTOR 5 (ARF5 [MP]), ARF3, and ARF4 are involved in regulating vascular development [44,45]. We detected no direct interaction between CsIVP and ARFs (S3 Fig); however, CsIVP did directly bind to the promoter region of the SCF^TIR1/AFB^ component, *CsAUX4*, and promoted its expression (Fig 3C, 3D, 3E and 3F). Previous work has shown that the SCF^TIR1/AFB^-mediated signaling pathway is involved in feedback regulation of IAA, through repressing auxin biosynthesis [46]. Therefore, CsIVP perhaps regulates the IAA level though the AUX4-mediated signaling pathway to mediate vascular patterning and organ morphogenesis in cucumber (Fig 7A).

Hormones play key roles in regulation of defense responses [47], and SA is an important activator of defense against biotrophic pathogens [48]. The observed resistance phenotype of *CsIVP*-RNAi plants to downy mildew led us to speculate that hormone pathways may be involved in CsIVP-mediated pathogen resistance (Fig 5). Consistently, we observed elevated levels of SA in *CsIVP*-RNAi leaf veins, along with transcript changes of marker genes, in this defense-related signaling pathway (Fig 6).

Upon infection by *P*. *cubensis*, *CsIVP* expression was rapidly reduced in WT (Fig 5I). Our interaction analysis confirmed that CsIVP directly interacts with CsNIMIN1, a repressor in the SA signaling pathway (Fig 6D and 6E). These findings suggest that CsIVP may act as a repressor for SA production and physically interacts with CsNIMIN1 to block the SA-responsive pathway to compromise downy mildew resistance in cucumber (Fig 7B). CsIVP may act as a positive regulator of CsNIMIN1 function. In the absence of CsIVP, the repressive role of CsNIMIN1 on SA response was compromised, which resulted in enhanced SA responses and elevated downy mildew resistance (Figs 5 and 6). To understand the mechanism of how CsIVP regulates CsNIMIN1 function, we overexpressed CsNIMIN1-HEMAGGLUTININ EPITOPE (HA) in protoplasts of WT and R5 transgenic plants. Our data showed that CsNIMIN1 accumulation was unchanged in R5 plants compared to WT (S6D Fig), indicating that CsIVP did not affect CsNIMIN1 protein stability. Future studies are in need to explore whether and how CsIVP affects the interaction of CsNIMIN1 with CsNPR1 and CsTGA, or the subcellular localization of CsNIMIN1 protein in cucumber.

JA plays a positive role in plant resistance against necrotrophic pathogens and herbivorous insects [48]. Our transcriptomics data showed that genes involved in the JA signaling pathway were significantly up-regulated in *CsIVP*-RNAi plants (S5B and S5C Fig). Considering that in the greenhouse *CsIVP*-RNAi plants exhibited resistance to several pathogens, in addition to downy mildew, we speculate that CsIVP may also participate in resistance to necrotrophic pathogens through the JA-signaling pathway. Future studies are needed to dissect the specific link between CsIVP and JA-mediated disease resistance in cucumber.

In *Arabidopsis*, *IND* and *HEC3* are paralogous genes of *CsIVP* that do not display roles in vasculature patterning or disease resistance [28,29]. Such functional divergence of *IND* and *HEC3* is probably due to neofunctionalization, after duplication. Both *IND* and *HEC3* function through the auxin signaling pathway. More importantly, *IND* regulates marginal tissue lignification, and lignin is required for xylem differentiation [28,49]. Given the early origin of *HEC* genes in mosses (S1A Fig), their conservation in relation to the vasculature, and their functional interaction with auxin signaling, we speculate that *HEC* genes were likely involved in emergence of the early land plant vascular system and, subsequently, in the development of more complex vascular configurations. It will be interesting to investigate whether *CsIVP* orthologs function by similar mechanisms for regulating immunity to pathogens and organ morphogenesis.

## Materials and methods

### Plant materials and growth conditions

Cucumber (*Cucumis sativus* L.) inbred line R1461, having a very similar genome to the sequenced variety Chinese Long 9930, was used in this study. Seeds were germinated, in the dark and at 28°C, and then grown in a growth chamber under a 16 h/8 h and 25°C/18°C day/night regime until the two true-leaf stage. Seedlings were then transferred to a standard greenhouse in the experimental field of China Agricultural University, Beijing. Water management and pest control were performed according to standard protocols.

### Gene cloning and phylogenetic analysis

The full-length coding sequence of *CsIVP* was cloned from line R1461. Total RNA was extracted from cucumber female buds, using a Quick RNA isolation Kit (Huayueyang Biotechnology, Beijing, China), and cDNA was synthesized using the TianScript II RT Kit (Tiangen Biotech, Beijing, China). Gene structure analysis of *CsIVP* was performed using the GSDS program (http://gsds.cbi.pku.edu.cn/). To retrieve protein and DNA sequences for phylogenetic analysis, *Arabidopsis* HEC1/2/3/IND and CsIVP were used as queries to search against the genome database, Phytozome (http://www.phytozome.net/), using BLASTP with a parameter of 1 × 10^−10^. Watermelon sequences were obtained from the Cucurbit Genomics Database (http://www.icugi.org/cgi-bin/ICuGI/index.cgi). Sequences of *Cucumis melo* and *Elaeis guineensis* were retrieved from the National Center for Biotechnology Information (NCBI; https://www.ncbi.nlm.nih.gov/). Gymnosperm sequences were collected from the OneKP transcriptome database (http://db.cngb.org/blast4onekp/). Accession numbers of all sequences used for phylogenetic analysis are provided in S7 Table. Full-length protein sequences of sampled species were aligned using the program Muscle 3.6 (http://www.drive5.com/muscle/), and the resulting alignment was manually adjusted in GeneDoc 3.2 [50]. Based on the protein alignment, a DNA matrix was produced by PAL2NAL (http://www.bork.embl.de/pal2nal/). The bHLH domain was identified according to the BoxShade program (http://www.ch.embnet.org/software/BOX_form.html). A phylogenetic tree was generated using the Neighbor-joining method and the bootstrap analysis with 1,000 replications in the MEGA6.0 software package [51].

### qRT-PCR

Total RNA was extracted from different cucumber tissues using a Quick RNA isolation Kit (Huayueyang Biotechnology, Beijing, China) and cDNA synthesized using a TianScript II RT Kit (Tiangen Biotech, Beijing, China). An ABI PRISM 7500 Real-Time PCR System (Applied Biosystems, Waltham, MA) was used for qRT-PCR assays. Cucumber *UBIQUITIN* (Csa000874) was used as internal references to normalize expression data. Each qRT-PCR experiment was performed with 3 biological and 3 technical replicates. Primer information is listed in S6 Table.

### In situ hybridization

Cucumber shoot apex, flower buds, and young fruits were fixed in 3.7% formal-acetic-alcohol (FAA) and in situ hybridization was performed, as previously described [30]. The *CsIVP* and *CsYAB5* probe was designed according to gene-specific fragments. Sense and antisense probes were generated by PCR amplification, with specific primers, using SP6 and T7 polymerase, respectively. Primers are listed in S6 Table.

### Subcellular localization assays

The *CsIVP* coding sequence, without the termination codon, was fused with the *GFP* coding sequence, then inserted into the plasmid pUC-SPYNE. Subcellular localization in onion epidermal cells was performed following standard protocols [49], and images were taken using a confocal laser-scanning microscope (Carl Zeiss LSM 510, Germany) excited at a 488 nm wavelength. Primers are listed in S6 Table.

### Cucumber transformation

To generate RNAi constructs, 270-bp gene-specific sense and antisense fragments of *CsIVP* and 248-bp of *CsYAB5* were amplified, using primers containing SpeI (5' end)/BamHI (3' end) and AscI (5' end)/SwaI (3' end) sites, respectively. The two fragments were cloned into the PFGC-1008 vector, with an empty PFGC-1008 vector used as a transformation control. The resultant *CsIVP*-RNAi and *CsYAB5*-RNAi constructs and the empty PFGC-1008 vector were introduced into *Agrobacterium* by electroporation. *CsIVP*-RNAi and *CsYAB5*-RNAi transgenic cucumber plants were obtained through a well-established cotyledon transformation method [30]. Primers are listed in S6 Table.

### Cytohistological analyses

Periodic acid-Schiff’s reagent (PAS) staining was used to analyze vascular cytohistological structure, which was performed on semi-thin sectioned leaf veins embedded in LR white resin, as previously described [52].

### Immunoblot

Full-length *CsIVP* CDS was cloned into the pET-28a vector to express CsIVP protein that was used to generate the polyclonal CsIVP antisera in rabbits, and the anti-CsIVP was tested through ELISA by the HUABIO HangZhou Company. Total protein was extracted from leaf tissues of WT and *CsIVP*-RNAi transgenic plants with extraction buffer (50 mM HEPES [pH 7.5], 150 mM KCl, 1 mM EDTA, 1 mM DTT, 0.5% Triton X-100, 1× protease inhibitors [Roche]), and the level of CsIVP was detected by anti-CsIVP immunoblots. Equal loading was shown by ponceau staining. The indicated plasmids were transfected into cucumber protoplasts and incubated overnight. Total protein was extracted using the aforementioned protocol. The protein level of NIMIN1-HA was detected by immunoblot, using the HA antibodies. The Luciferase protein was used as a control.

### Leaf decolorization protocol

Cucumber leaves were bathed overnight in a mixture of 14% acetic acid and 86% ethyl alcohol. Leaves were then dehydrated in 70% ethyl alcohol, twice, for 5 min and then transferred to a clearing solution (80% lactic acid to phenol to glycerin to distilled water at 1:1:1:1) in a 90°C water bath. The resultant transparent leaves were imaged with a scanner (Epson perfection V800 photo, Canada).

### Triphenyltetrazolium chloride test

To examine viability, pollen was placed on a glass slide and then incubated for 15 min at 37°C with a 0.5% triphenyltetrazolium chloride (TTC) solution (0.5 g TTC dissolved in 100 ml distilled water); viable pollen grains were stained red. Images were photographed using a light microscope (D72, Olympus, Tokyo, Japan). Five plants from each *CsIVP-*RNAi transgenic line and WT were selected, 3 flowers per plant were harvested, 5 slides were prepared for each flower, and 3 fields per slide were examined. Pollen viability was calculated as the ratio of red pollen grains to total pollen grains.

### Measurements of endogenous hormones

To examine the levels of auxin (IAA), ZR, GA, ABA, and BRs in WT and *CsIVP-*RNAi transgenic plants, approximately 0.1 g samples were harvested from leaf veins. Hormone analyses were performed using ELISA, after sample extraction, as previously described [53]. To examine the level of SA, approximately 25 mg samples were vacuum freeze-dried for 10 to 12 h, followed by dipping into cold buffer 1 (MeOH:H_2_O = 9:1, 0.1% formic acid, 0.05 g lidocaine, 10 ng D6ABA) for 12 h in 4°C. After centrifuging two times, the supernatants were dried with nitrogen and then dissolved with 100 μl methyl alcohol. The new supernatant concentration was detected by Agilent 6520. Three biological replicates were performed for each tissue type.

### IAA immunolocalization

Young leaf veins, and fruits of WT and *CsIVP-*RNAi transgenic cucumber plants, were cross-linked in pre-cooled 3% EDC for 1 h in a dark chamber, at 4°C, and then kept in paraformaldehyde for overnight fixation. Fixed samples were dehydrated, embedded, sectioned, dewaxed, and then incubated, overnight, with anti-IAA monoclonal antibodies [54]. The Dylight 488 antibodies were applied on slides and incubated for 4 h at room temperature in the dark. After washing with 10 mM phosphate buffered saline (PBS), twice for 5 min each, specimens were mounted in 50% glycerin and imaged under a confocal laser-scanning microscope (Carl Zeiss LSM 510, Germany).

### RNA-seq and bioinformatics analyses

Leaf veins (midveins dissected from the youngest expanded leaves) and young fruits (4 d before anthesis) of WT and transgenic cucumber plants were collected for RNA-seq analysis. Two biological replicates were performed for each tissue sampled. RNA-seq library construction was performed using the NEBNext Ultra Directional RNA Library Prep Kit for Illumina (NEB, Ispawich, MA), according to the manufacturer’s instructions, and 4 index codes were added to attribute sequences to different samples [55]. The Illumina HiSeq 2000 platform was used to sequence the RNA-seq libraries to generate 150 bp paired-end reads. Sequencing data were deposited with the Gene Expression Omnibus (GEO) database at the NCBI under accession number GSE86496.

Analyses of RNA-seq data were performed, as previously described [53]. Reads longer than 25 bp were mapped to the cucumber genome sequence (http://cucumber.genomics.org.cn, version 2i), using TopHat [56,57]. The read counts of annotated genes were counted using HTSeq-count [58]. The genes with a normalized expression level of at least 1 transcript per million (TPM), in at least 2 samples, were retained for further analysis. The R package, edgeR, was used to identify DEGs between WT and *CsIVP-*RNAi transgenic plants [59]. The cutoff for DEGs was at least a 2-fold change in expression and a false discovery rate (FDR) of less than 0.05.

### Yeast one-hybrid assays

Sense and antisense oligonucleotides were obtained by synthesizing 2 antiparallel oligonucleotides containing a triplication of each E-box variant, plus 3 nucleotides of the flanking sequence on both sides, based on the promoters of *CsAUX4*, *CsYAB5*, and *CsBP*. A mutated E-box was used as a negative control. Then, sense and antisense nucleotides were cloned into the pAbAi vector (Clontech, Mountain View, CA); the resultant plasmids and an empty pAbAi were transformed into the yeast strain Y1H Gold, according to the manufacturer’s instructions. The full-length coding sequence of CsIVP, CsHEC1, or CsHEC2 was cloned into pGADT7, and then the plasmid was transformed into the Y1H Gold strain, with pE-box-ABAi. Positive colonies were selected by Aureobasidin A (AbA) on Synthetic Dropout (SD)/Leucine (Leu) medium (Clontech). Primer information for oligonucleotide synthesis is listed in S6 Table.

### EMSAs

The full-length coding sequence of *CsIVP* was cloned into the pET-28a vector, sequenced, and transformed into the BL21 *Escherichia coli* strain for protein expression. When the culture OD_600_ was 0.6–0.8, IPTG was added for protein induction for 16 h at 16°C. Proteins were extracted and purified using Ni-NTA and analyzed by SDS-PAGE. DNA fragments, approximately 35 to 40 bp in length—containing the E-box of the *CsBP*, *CsAUX*, *CsYAB5*, or *CsCCR1* promoters—were labeled with biotin and used as probes; cold probes (unlabeled probes) were used as competitors. EMSA assays were performed with a LightShift Chemiluminescent EMSA Kit (Thermo #20148), according to the manufacturer’s instructions. Probe sequences are listed in S6 Table.

### ChIP-PCR

ChIP-PCR was performed as previously described [60]. Approximately 2 g floral and leaf tissues from control and transgenic lines were used to harvest sonicated chromatin. An anti-myc antibody (Abcam anti-Myc tag antibody, lot number GR310953-4) was used to perform immunoprecipitation reactions. Three biological repeats and three technical replicates were performed for each sequence segment. *CsTUBLIN* was used as the internal control. The primer information used in ChIP-PCR is listed in S6 Table.

### LUC/REN assay for protein–DNA interactions in tobacco leaves

Promoters of *CsYAB5* (1,766 bp), *CsBP* (1,816 bp), and *CsAUX4* (1,907 bp) were cloned into the transient expression vector pGreenII 0800-LUC as reporters. The CDS of CsIVP, CsHEC1, or CsHEC2 was cloned into pGreenII 62-SK as effectors. The agrobacterium GV3101 strain carrying the earlier-verified constructs and the *N*. *benthamiana* leaves were used to detect the co-expression, as previously described [61]. Six biological repeats were used to calculate the ratio of LUC to REN, which reflected the final transcriptional activity. The primers for all constructs are listed in S6 Table.

### Phytopathological tests and disease evaluation

WT and *CsIVP*-RNAi (R5) seedlings were grown in a growth chamber at 22°C with 12 h light/dark photoperiod. When the sixth true leaf was fully expanded, plants were inoculated with 3 × 10^5^ *P*. *cubensis* sporangia/ml solution by spraying the leaf surface with the same droplet density. After inoculation, plants were kept in darkness for 24 h at 100% relative humidity and 19°C, and then moved to a 12 h/12 h light/dark cycle, with 85% relative humidity and 19°C. The rating assessments of foliar disease were performed 11 dpi, using a scale of 0 to 9, where 0 = no disease, 1 = the area of lesion is less than 3% of the whole leaf area, 2 = approximately 3%–6%, 3 = approximately 6%–12%, 4 = approximately 12%–25%, 5 = approximately 25%–50%, 6 = approximately 50%–70%, 7 = approximately 75%–87%, 8 = approximately 87%–100%, and 9 = 100% [62]. The disease index was calculated according to the formula: $X=\frac{\Sigma (Ni\times i)}{N\times 9}\times 100$. X indicates the disease index, Ni indicates the number of leaves with different disease indices, i represents the numerical value of different disease indices, and *N* indicates the total number of leaves. Each treatment had 3 biological replicates, with 5 plants in each replicate.

### Scanning electron microscope

Disease leaf samples were fixed in FAA for 4 h. After critical-point drying, samples were sputter-coated with gold and examined on a scanning electron microscope, using an acceleration voltage of 2 kV.

### Y2H assays

Full-length coding sequences for the specified genes, including CsIVP, CsHEC1, CsHEC2, and CsNIMIN1, were cloned into pGADT7 (bait vector) or pGBKT7 (prey vector), sequenced, and transformed into the yeast strain AH109. The bait and prey vectors were transformed, following the instructions for the MatchmakerTM GAL4 Two-Hybrid System 3 & Libraries (Clontech). Protein interactions were assayed, as previously described [63]. Primers for the Y2H assays are listed in S6 Table.

### BiFC assays

Full-length coding sequences for CsIVP, CsHEC1, CsHEC2, and CsNIMIN1 were amplified by PCR, without stop codons, and cloned into pSPYNE-35S and pSPYCE-35S vectors containing the N- or C- terminus of YFP to generate in-frame fusion proteins. All constructs were confirmed by sequencing and then transformed into the *A*. *tumefaciens* strain GV3101. The 2 plasmids for detection of specific protein interactions were co-transformed into the abaxial side of 5- to 6-wk-old tobacco (*N*. *benthamiana*) leaves, as previously described [63]. Tobacco leaves were examined using a Zeiss LSM 510 Meta confocal laser-scanning microscope, after 48-h co-infiltration. YFP signals were imaged under 488 nm excitation wavelength. Primers used for BiFC are listed in S6 Table.

## Supporting information

S1 Fig Phylogenetic relationship among HEC genes and expression analysis of *CsIVP* in cucumber. (A) The diagram shows there was a duplication before appearance of the angiosperms. Bootstrap values over 50% are placed above the branches. (B–E) qRT-PCR analyses of *CsIVP* in different organs of cucumber (panels B–D) and in different tissues of young fruit (panel E). The *UBIQUITIN* (*Csa000874*) gene was used as an internal control to normalize expression. Values are means ± SE (n = 3). Numbers 1, 5, 8, 10, and 15 of leaf and node represent nodes from bottom to top, and “F” represent fruit. (F) Subcellular localization of CsIVP in onion epidermal cells. GFP is indicated by green fluorescent signal. GFP driven by the *CaMV 35S* promoter was used as a control. Scale bars represent 50 μm in panel F. The data underlying this figure are included in S6 Data.
(TIF)

S2 Fig Phenotypic characterization and hormone measurements performed on *CsIVP*-RNAi transgenic plants. (A–B) Expression of *CsHEC1* and *CsHEC2* was unchanged in the *CsIVP*-RNAi lines. Values are means ± SE (*n* = 3). (C–D) Reduced plant height and increased stem diameter in *CsIVP-*RNAi transgenic plants. Red brackets indicated the second node in WT and *CsIVP*-RNAi line R5. (E–F) Quantification of plant height (panel E) and stem diameter (panel F). WAG, weeks after germination. Values are means ± SE (*n* = 3). (G) Corolla diameter of the male flower. Values are means ± SE (*n* = 10). (H-J) Quantification of ovary diameter (panel H) and length (panel I) at anthesis, and mature fruit length (panel J) in WT and *CsIVP*-RNAi lines. (K) Stamens at anthesis. (K’) Enlarged views of red boxes in panel K. Released pollen grains are bright spots (red stars). (L) Pollen viability assay. Viable pollen grains are stained red. (M) Morphological characterization of mature seeds of WT and *CsIVP*-RNAi lines. (N–O) Transverse sections of fruit in WT and *CsIVP*-RNAi R5 line. White numbers indicate the vascular bundles. (P) Content of ZR, GA3, BRs, and ABA in the leaf veins of WT and *CsIVP*-RNAi plants. (Q–T) IAA distribution in young fruits, as detected by immunolocalization. Anti-IAA monoclonal antibodies and DyLight 488–conjugated goat anti-mouse IgG antibodies were used to detect IAA. (Q, S) Differential interference contrast images, and (R, T) fluorescent images. Scale bars represented 2 cm in panels C–D and M; 500 μm in panels K–L and O; 50 μm in panel K’; 2 mm in panel N; and 100 μm in panels Q–T. Values are means ± SE (*n* = 3) in panels H–J and P. Asterisk and double asterisks in panels G–J indicate significant differences of *P* < 0.05 and *P* < 0.01 by *t* test, respectively. The data underlying this figure are included in S7 Data. ABA, abscisic acid; BR, brassinosteroid; GA3, gibberellic acid3; ZR, zeatin riboside
(TIF)

S3 Fig Expression analyses and interaction summary of genes involved in vasculature development. (A) qRT-PCR verification of DEGs identified by RNA-seq analysis. The *UBIQUITIN (Csa000874)* gene was used as an internal control to normalize expression levels. Values are means ± SE (*n* = 3), double asterisks indicate significant difference at *P* < 0.01 by *t* test. (B) Summary of yeast one-hybrid assays performed in this study. “+” indicates positive interaction, “−” indicates no interaction, “Un” indicates untested, “*” represents confirmed by EMSA, ChIP-PCR, and luciferase activity. (C) Yeast one-hybrid assay between CsHEC1/2 and the E-box from the *CsYAB5*, *CsBP*, and *CsAUX4* promoters. The SD/-Leu medium with 100 ng/ml or 500 ng/ml inhibitory AbA was used to screen for interactions. (D–F) Luciferase activity measured in tobacco leaves after co-expression of *35S*:*CsHEC1/2* with *proCsYAB5*:*LUC*, or *proCsBP*:*LUC*, or *proCsAUX4*:*LUC*. Values are means ± SE (*n* = 6). The data underlying this figure are included in S8 Data. AbA, Aureobasidin A
(TIF)

S4 Fig *CsYAB5* expression analyses in different cucumber organs. (A–C) qRT-PCR analyses of *CsYAB5* in leaf (panel A), stem (panel B), and fruit (panel C). The *UBIQUITIN* (*Csa000874*) gene was used as an internal control to normalize expression. Values are means ± SE (*n* = 3). Numbers 1, 5, 8, 10, and 15 of leaf and stem represent nodes from bottom to top; “F” represent fruit. The data underlying this figure are included in S6 Data.
(TIF)

S5 Fig Disease index and transcriptome analysis in WT and *CsIVP*-RNAi transgenic plants. (A) Disease index of WT and *CsIVP*-RNAi plants under greenhouse and growth chamber conditions. (B) MapMan analysis of DEGs in veins of *CsIVP*-RNAi transgenic compared to WT plants that are involved in the response to biotic stresses. (C) Bar plot showing the top 5 GO terms in the up- and down-regulated DEGs in the leaf vein of *CsIVP* transgenic plants compared to WT. The data underlying this figure are included in S9 Data.
(TIF)

S6 Fig Interactions detected by Y2H, BiFC, and immunoblots. (A) Summary of protein interactions of disease resistance performed in this study. “++” indicates strong interaction, “+” indicates positive interaction, “*” represents confirmed by BiFC, “–” indicates no interaction; “Un” indicates untested. CsIVP-BD indicates CsIVP fused with the GAL4 DNA binding domain. AtHAN-AD denotes AtHAN fused with the activation domain. Similar labels were used for the other constructs. (B) Y2H assays. A combination of AtHAN-BD and AtHAN-AD was used as the positive control [63]; the combinations of each gene and the empty vectors pGBKT7 and pGADT7 were used as negative controls. (C) BiFC assays. IND-YFPC and SPT-YFPN were used as positive controls [49]. Protein interactions are indicated by green YFP fluorescent signals in nuclei (left panels); DIC images of tobacco cells are shown in the middle panels; and merged channels are shown in right panels. (D) Immunoblot of protoplasts from WT and R5 transgenic plants overexpressing CsNIMIN1-HA. AD, activation domain; BD, binding domain; DIC, differential interference contrast; IND, INDEHISCENT; SPT, SPATULA; Y2H, yeast two-hybrid
(TIF)

S1 Table Summary of transcriptome sequencing data. (DOCX)

S2 Table RNA-seq data. (XLSX)

S3 Table Examples of genes involved in vascular development that are differentially expressed in the veins of R5 versus WT in cucumber. (DOCX)

S4 Table Examples of auxin-related genes differentially expressed in the veins of R5 versus WT in cucumber. (DOCX)

S5 Table Summary of CsIVP binding to the E-box in promoters and introns of *CsYAB5*, *CsBP*, *CsAUX4*, and *CsCCR1* genes. (DOCX)

S6 Table Primers used in this study. (DOCX)

S7 Table Gene accession numbers used for phylogenetic analysis. (DOCX)

S1 Data The data underlying Fig 2. (XLSX)

S2 Data The data underlying Fig 3. (XLSX)

S3 Data The data underlying Fig 4. (XLSX)

S4 Data The data underlying Fig 5. (XLSX)

S5 Data The data underlying Fig 6. (XLSX)

S6 Data The data underlying S1 and S4 Figs. (XLSX)

S7 Data The data underlying S2 Fig. (XLSX)

S8 Data The data underlying S3 Fig. (XLSX)

S9 Data The data underlying S5 Fig. (XLSX)

S1 Raw Images Original images for blots and gels in Figs 2C and 3D and S6D Fig. (PDF)

## Abbreviations

AbA: aureobasidin A
ABA: abscisic acid
APL: ALTERED PHLOEM DEVELOPMENT
ARF: AUXIN RESPONSE FACTOR
as2: ASYMMETRIC LEAVES2
AUX4: AUXIN/INDOLEACETIC ACIDS4
bHLH: basic Helix-Loop-Helix
BiFC: bimolecular fluorescence complementation
BP: BREVIPEDICELLUS
BR: brassinosteroid
CCR1: CINNAMOYL COA REDUCTASE1
ChIP: chromatin immunoprecipitation
CsIVP: Cucumis sativus Irregular Vasculature Patterning
DEG: differentially expressed gene
dpi: days post inoculation
EMSA: electrophoretic mobility-shift assay
FAA: formal-acetic-alcohol
FDR: false discovery rate
FIL: FILAMENTOUS FLOWER
GA3: gibberellic acid3
GEO: Gene Expression Omnibus
GFP: green fluorescent protein
GO: gene ontology
HA: HEMAGGLUTININ EPITOPE
HEC3: HECATE3
IAA: indole-3-acetic acid
IND: INDEHISCENT
IRX: IRREGULAR XYLEM
ITS: internal transcribed spacer
JA: jasmonic acid
KAN: KANADI
KNAT7: KNOTTED1-LIKE HOMEOBOX GENE 7
LUC/REN: luciferase/renilla
MYB: myeloblastosis
MYC: c-myc
NAC: NAM/ATAF/CUC
NCBI: National Center for Biotechnology Information
NIMIN1: NIM1-INTERACTING1
NPR: NONEXPRESSOR OF PATHOGENESIS-RELATED GENES 1
OPS: OCTOPUS
OsYAB4: *Oryza sativa* YABBY4
PAS: Periodic acid-Schiff’s reagent
PBS: phosphate buffered saline
PHB: PHABULOSA
PR1: PATHOGENESIS-RELATED PROTEIN1
qRT-PCR: quantitative real-time PCR
RNA-seq: RNA sequencing
RNAi: RNA interference
SA: salicylic acid
SNI1: SUPPRESSOR OF NPR1
TPM: transcripts per million
TTC: triphenyltetrazolium chloride
VND7: VASCULAR-RELATED NAC-DOMAIN 7
WHY1: WHIRLY 1
WRKY: DNA-binding domain containing a highly conserved WRKYGQK motif
WT: wild type
Y2H: yeast two-hybrid
YAB: YABBY
YFP: yellow fluorescent protein
ZR: zeatin riboside

## References

[1] Ladizinsky G. Plant evolution under domestication: Springer Netherlands; 1998.

[2] Stenberg JA, Heil M, Ahman I, Bjorkman C. Optimizing crops for biocontrol of pests and disease. Trends Plant Sci. 2015; 20: 698–712. 10.1016/j.tplants.2015.08.007 26447042

[3] Tian L, Shi S, Nasir F, Chang C, Li W, Tran LP, et al Comparative analysis of the root transcriptomes of cultivated and wild rice varieties in response to *magnaporthe oryzae* infection revealed both common and species-specific pathogen responses. Rice (N Y). 2018; 11: 26. 29679239 10.1186/s12284-018-0211-8 PMC5910329

[4] Brown JK. Yield penalties of disease resistance in crops. Curr Opin Plant Biol. 2002; 5: 339–344. 10.1016/s1369-5266(02)00270-4 12179968

[5] Karasov T, Chae E, Herman J, Bergelson J. Mechanisms to mitigate the tradeoff between growth and defense. Plant Cell. 2017; 29: 666 10.1105/tpc.16.00931 28320784 PMC5435432

[6] Qi J, Liu X, Shen D, Miao H, Xie B, Li X, et al A genomic variation map provides insights into the genetic basis of cucumber domestication and diversity. Nat Genet. 2013; 45: 1510–1515. 10.1038/ng.2801 24141363

[7] Herraiz FJ, Raigon MD, Vilanova S, Garcia-Martinez MD, Gramazio P, Plazas M, et al Fruit composition diversity in land races and modern pepino (*solanum muricatum*) varieties and wild related species. Food Chem. 2016; 203: 49–58. 10.1016/j.foodchem.2016.02.035 26948588

[8] Lucas WJ, Groover A, Lichtenberger R, Furuta K, Yadav SR, Helariutta Y, et al The plant vascular system: evolution, development and functions. J Integr Plant Biol. 2013; 55: 294–388. 10.1111/jipb.12041 23462277

[9] Ye ZH. Vascular tissue differentiation and pattern formation in plants. Plant Biology. 2002; 53: 183–202. 10.1146/annurev.arplant.53.100301.135245 12221972

[10] HU L, SUN H, LI R, Zhang L, Wang S, Sui X, et al Phloem unloading follows an extensive apoplasmic pathway in cucumber (*Cucumis sativus* L.) fruit from anthesis to marketable maturing stage. Plant Cell & Environment. 2011; 34: 1835–1848. 10.1111/j.1365-3040.2011.02380.x 21707653

[11] Scarpella E, Helariutta Y. Vascular pattern formation in plants. Curr Top Dev Biol. 2010; 91: 221–265. 10.1016/S0070-2153(10)91008-9 20705184

[12] Yamaguchi M, Mitsuda N, Ohtani M, Ohme-Takagi M, Kato K, Demura T. VASCULAR-RELATED NAC-DOMAIN7 directly regulates the expression of a broad range of genes for xylem vessel formation. Plant J. 2011; 66: 579–590. 10.1111/j.1365-313X.2011.04514.x 21284754

[13] Zhong R, Lee C, Zhou J, Mccarthy RL, Ye ZH. A battery of transcription factors involved in the regulation of secondary cell wall biosynthesis in Arabidopsis. Plant Cell. 2008; 20: 2763–2782. 10.1105/tpc.108.061325 18952777 PMC2590737

[14] Smith HM, Hake S. The interaction of two homeobox genes, BREVIPEDICELLUS and PENNYWISE, regulates internode patterning in the Arabidopsis inflorescence. Plant Cell. 2003; 15: 1717–1727. 10.1105/tpc.012856 12897247 PMC167164

[15] Bonke M, Thitamadee S, Mahonen AP, Hauser MT, Helariutta Y. APL regulates vascular tissue identity in Arabidopsis. Nature. 2003; 426: 181–186. 10.1038/nature02100 14614507

[16] Anne Pauline, Azzopardi Marianne, Gissot Lionel, et al OCTOPUS negatively regulates BIN2 to control phloem differentiation in Arabidopsis thaliana. Current Biology. 2015; 25: 2584–2590. 10.1016/j.cub.2015.08.033 26387715

[17] Dengler N, Kang J. Vascular patterning and leaf shape. Current Opinion in Plant Biology. 2001; 4: 50–56. 10.1016/s1369-5266(00)00135-7 11163168

[18] Feng S, Xu Y, Guo C, Zheng J, Zhou B, Zhang Y, et al Modulation of miR156 to identify traits associated with vegetative phase change in tobacco (*nicotiana tabacum*). Journal of Experimental Botany. 2016; 67: 1493 10.1093/jxb/erv551 26763975

[19] Nelson T, Dengler N. Leaf vascular pattern formation. Plant Cell. 1997; 9: 1121–1135. 10.1105/tpc.9.7.1121 12237378 PMC156985

[20] Eshed Y, Izhaki A, Baum SF, Floyd SK, Bowman JL. Asymmetric leaf development and blade expansion in Arabidopsis are mediated by KANADI and YABBY activities. Development. 2004; 131: 2997–3006. 10.1242/dev.01186 15169760

[21] McConnell JR, Emery J, Eshed Y, Bao N, Bowman J, Barton MK. Role of PHABULOSA and PHAVOLUTA in determining radial patterning in shoots. Nature. 2001; 411: 709–713. 10.1038/35079635 11395776

[22] Carella P, Wilson DC, Kempthorne CJ, Cameron RK. Vascular sap proteomics: providing insight into long-distance signaling during stress. Front Plant Sci. 2016; 7: 651 10.3389/fpls.2016.00651 27242852 PMC4863880

[23] Vlot AC, Dempsey DA, Klessig DF. Salicylic acid, a multifaceted hormone to combat disease. Annual Review of Phytopathology. 2009; 47: 177–206. 10.1146/annurev.phyto.050908.135202 19400653

[24] Kachroo A, Kachroo P. Salicylic acid-, jasmonic acid- and ethylene-mediated regulation of plant defense signaling. Genet Eng (N Y). 2007; 28: 55–83. 17153933 10.1007/978-0-387-34504-8_4

[25] Weigel RR, Pfitzner UM, Gatz C. Interaction of NIMIN1 with NPR1 modulates PR gene expression in Arabidopsis. Plant Cell. 2005; 17: 1279–1291. 10.1105/tpc.104.027441 15749762 PMC1088002

[26] Vos IA, Pieterse CMJ, Van Wees SCM. Costs and benefits of hormone-regulated plant defences; 2013; pp. 43–55.

[27] Zhao J, Li Y, Ding L, Yan S, Liu M, Jiang L, et al Phloem transcriptome signatures underpin the physiological differentiation of the pedicel, stalk and fruit of cucumber (*Cucumis sativus* L.). Plant Cell Physiol. 2016; 57: 19–34. 10.1093/pcp/pcv168 26568324

[28] Gremski K, Ditta G, Yanofsky MF. The HECATE genes regulate female reproductive tract development in Arabidopsis thaliana. Development. 2007; 134: 3593–3601. 10.1242/dev.011510 17855426

[29] Liljegren SJ, Roeder AH, Kempin SA, Gremski K, Ostergaard L, Guimil S, et al Control of fruit patterning in Arabidopsis by INDEHISCENT. Cell. 2004; 116: 843–853. 10.1016/s0092-8674(04)00217-x 15035986

[30] Ding L, Yan S, Jiang L, Liu M, Zhang J, Zhao J, et al HANABA TARANU regulates the shoot apical meristem and leaf development in Cucumber (*Cucumis sativus* L.). Journal of Experimental Botany. 2015; 66: 7075 10.1093/jxb/erv409 26320238 PMC4765787

[31] Aloni R, Aloni E, Langhans M, Ullrich CI. Role of cytokinin and auxin in shaping root architecture: regulating vascular differentiation, lateral root initiation, root apical dominance and root gravitropism. Ann bot (lond). Annals of Botany. 2006; 97: 883–893. 10.1093/aob/mcl027 PMC2803412 16473866

[32] De Rybel B, Mahonen AP, Helariutta Y, Weijers D. Plant vascular development: from early specification to differentiation. Nat Rev Mol Cell Biol. 2016; 17: 30–40. 10.1038/nrm.2015.6 26580717

[33] Toledo-Ortiz G, Huq E, Quail PH. The Arabidopsis Basic/helix-loop-helix transcription factor family. Plant Cell. 2003; 15: 1749–1770. 10.1105/tpc.013839 12897250 PMC167167

[34] Strable J, Wallace JG, Unger-Wallace E, Briggs S, Bradbury P, Buckler ES, et al Maize YABBY genes drooping leaf1 and drooping leaf2 regulate plant architecture. Plant Cell. 2017; 29: 477–2016. 10.1105/tpc.16.00477 PMC5559738 28698237

[35] Stahle MI, Janine K, Lindsay S, Arnim AGV, Golz JF. YABBYs and the transcriptional corepressors LEUNIG and LEUNIG_HOMOLOG maintain leaf polarity and meristem activity in Arabidopsis. Plant Cell. 2009; 21: 3105–3118. 10.1105/tpc.109.070458 19837869 PMC2782291

[36] Savory EA, Granke LL, Quesada-Ocampo LM, Varbanova M, Hausbeck MK, Day B. The cucurbit downy mildew pathogen *pseudoperonospora cubensis*. Mol Plant Pathol. 2011; 12: 217–226. 10.1111/j.1364-3703.2010.00670.x 21355994 PMC6640371

[37] Tian M, Win J, Savory E, Burkhardt A, Held M, Brandizzi F, et al 454 genome sequencing of pseudoperonospora cubensis reveals effector proteins with a QXLR translocation motif. Mol Plant Microbe Interact. 2011; 24: 543 10.1094/MPMI-08-10-0185 21261462

[38] Siegfried K, Eshed YS, Otsuga D, Drews G, Bowman J. Members of the YABBY gene family specify abaxial cell fate in Arabidopsis. Development. 1999; 126: 4117 10457020 10.1242/dev.126.18.4117

[39] Golz JF, Mario R, Robert K, Andrew H. GRAMINIFOLIA promotes growth and polarity of Antirrhinum leaves. Development. 2004; 131: 3661 10.1242/dev.01221 15229175

[40] Yang C, Ma Y, Li J. The rice YABBY4 gene regulates plant growth and development through modulating the gibberellin pathway. Journal of Experimental Botany. 2016; 18: 5545. 10.1093/jxb/erw319 27578842

[41] Husbands AY, Benkovics AH, Nogueira FT, Lodha M, Timmermans MC. The ASYMMETRIC LEAVES complex employs multiple modes of regulation to affect adaxial-abaxial patterning and leaf complexity[open]. Plant Cell. 2015; 27: 3321 10.1105/tpc.15.00454 26589551 PMC4707451

[42] Semiarti E, Ueno Y, Tsukaya H, Iwakawa H, Machida C, Machida Y. The ASYMMETRIC LEAVES2 gene of Arabidopsis thaliana regulates formation of a symmetric lamina, establishment of venation and repression of meristem-related homeobox genes in leaves. Development. 2001; 128: 1771–1783. 11311158 10.1242/dev.128.10.1771

[43] Venglat SP, Dumonceaux T, Rozwadowski K, Parnell L, Babic V, Keller W, et al The homeobox gene BREVIPEDICELLUS is a key regulator of inflorescence architecture in Arabidopsis. Proc Natl Acad Sci U S A. 2002; 99: 4730–4735. 10.1073/pnas.072626099 11917137 PMC123716

[44] Hardtke CS, Berleth T. The Arabidopsis gene MONOPTEROS encodes a transcription factor mediating embryo axis formation and vascular development. EMBO J. 1998; 17: 1405–1411. 10.1093/emboj/17.5.1405 9482737 PMC1170488

[45] Williams L, Carles CC, Osmont KS, Fletcher JC, Zambryski PC. A database analysis method identifies an endogenous trans-acting short-interfering RNA that targets the Arabidopsis. Proceedings of the National Academy of Sciences. 2005; 102: 9703–9708. 10.1073/pnas.0504029102 PMC1172271 15980147

[46] Takato S, Kakei Y, Mitsui M, Ishida Y, Suzuki M, Yamazaki C, et al Auxin signaling through SCF^TIR1/AFBS^ mediates feedback regulation of IAA biosynthesis. Bioscience Biotechnology & Biochemistry. 2017; 81: 1–7. 10.1080/09168451.2017.1313694 28406060

[47] Caarls L, Pieterse CM, Van Wees SC. How salicylic acid takes transcriptional control over jasmonic acid signaling. Front Plant Sci. 2015; 6: 170 10.3389/fpls.2015.00170 25859250 PMC4373269

[48] Pieterse CM, Van DDD, Zamioudis C, Leonreyes A, Van Wees SC. Hormonal modulation of plant immunity. Annual Review of Cell & Developmental Biology. 2012; 28: 489. 10.1146/annurev-cellbio-092910-154055 22559264

[49] Girin T, Paicu T, Stephenson P, Fuentes S, Korner E, O'Brien M, et al INDEHISCENT and SPATULA interact to specify carpel and valve margin tissue and thus promote seed dispersal in Arabidopsis. Plant Cell. 2011; 23: 3641–3653. 10.1105/tpc.111.090944 21990939 PMC3229140

[50] Nicholas K, Nicholas H. GeneDoc: a tool for editing and annotating multiple sequence alignments. 1997; 4.

[51] Tamura K, Stecher G, Peterson D, Filipski A, Kumar S. MEGA6: molecular evolutionary genetics analysis version 6.0. Molecular Biology and Evolution. 2013; 30: 2725–2729. 10.1093/molbev/mst197 24132122 PMC3840312

[52] Xu TT, Ren SC, Song XF, Liu CM. CLE19 expressed in the embryo regulates both cotyledon establishment and endosperm development in Arabidopsis. Journal of Experimental Botany. 2015; 66: 5217–5227. 10.1093/jxb/erv293 26071532 PMC4526921

[53] Sun C, Li Y, Zhao W, Song X, Lu M, Li X, et al Integration of hormonal and nutritional cues orchestrates progressive corolla opening. Plant Physiol. 2016; 171: 1209–1229. 10.1104/pp.16.00209 27208289 PMC4902604

[54] Sakata T, Oshino T, Miura S, Tomabechi M, Tsunaga Y, Higashitani N, et al Auxins reverse plant male sterility caused by high temperatures. Proc Natl Acad Sci U S A. 2010; 107: 8569–8574. 10.1073/pnas.1000869107 20421476 PMC2889339

[55] Wang Z, Gerstein M, Snyder M. RNA-Seq: a revolutionary tool for transcriptomics. Nat Rev Genet. 2009; 10: 57–63. 10.1038/nrg2484 19015660 PMC2949280

[56] Trapnell C, Pachter L, Salzberg SL. TopHat: discovering splice junctions with RNA-Seq. Bioinformatics. 2009; 25: 1105–1111. 10.1093/bioinformatics/btp120 19289445 PMC2672628

[57] Huang S, Li R, Zhang Z, Li L, Gu X, Fan W, et al The genome of the cucumber, *Cucumis sativus* L. Nat Genet. 2009; 41: 1275–1281. 10.1038/ng.475 19881527

[58] Anders S, Pyl PT, Huber W. HTSeq—a python framework to work with high-throughput sequencing data. Bioinformatics. 2014; 31: 166–169. 10.1093/bioinformatics/btu638 25260700 PMC4287950

[59] Robinson MD, Mccarthy DJ, Smyth GK. edgeR: a Bioconductor package for differential expression analysis of digital gene expression data. Bioinformatics. 2010; 26: 139–140. 10.1093/bioinformatics/btp616 19910308 PMC2796818

[60] Zhao J, Jiang L, Che G, Pan Y, Zhang X. A functional allele of CsFUL1 regulates fruit length through inhibiting CsSUP and auxin transport in cucumber. The Plant Cell. 2019: 905–2018. 10.1105/tpc.18.00905 PMC6588310 30979795

[61] Liu X, Ning K, Che G, Yan S, Han L, Gu R, et al CsSPL functions as an adaptor between HD-ZIP III and CsWUS transcription factors regulating anther and ovule development in cucumber. Plant Journal for Cell & Molecular Biology. 2018; 94. 10.1111/tpj.13877 29474743

[62] Jenkins JA S. F., Wehner TC. A system for the measurement of foliar diseases of cucumber. Cucurbit Genet. Coop. Rpt. 1983: 10–12.

[63] Ding L, Yan S, Jiang L, Zhao W, Ning K, Zhao J, et al HANABA TARANU (HAN) bridges meristem and organ primordia boundaries through PINHEAD, JAGGED, BLADE-ON-PETIOLE2 and CYTOKININ OXIDASE 3 during flower development in Arabidopsis. PLoS Genet. 2015; 11: e1005479 10.1371/journal.pgen.1005479 26390296 PMC4577084

