# Supplementary figures and images for: CsIVP functions in vasculature development and downy mildew resistance in cucumber
Source: PLoS Biol. 2020 Mar 23;18(3):e3000671. doi: 10.1371/journal.pbio.3000671 (PMC7117775; doi:10.1371/journal.pbio.3000671)

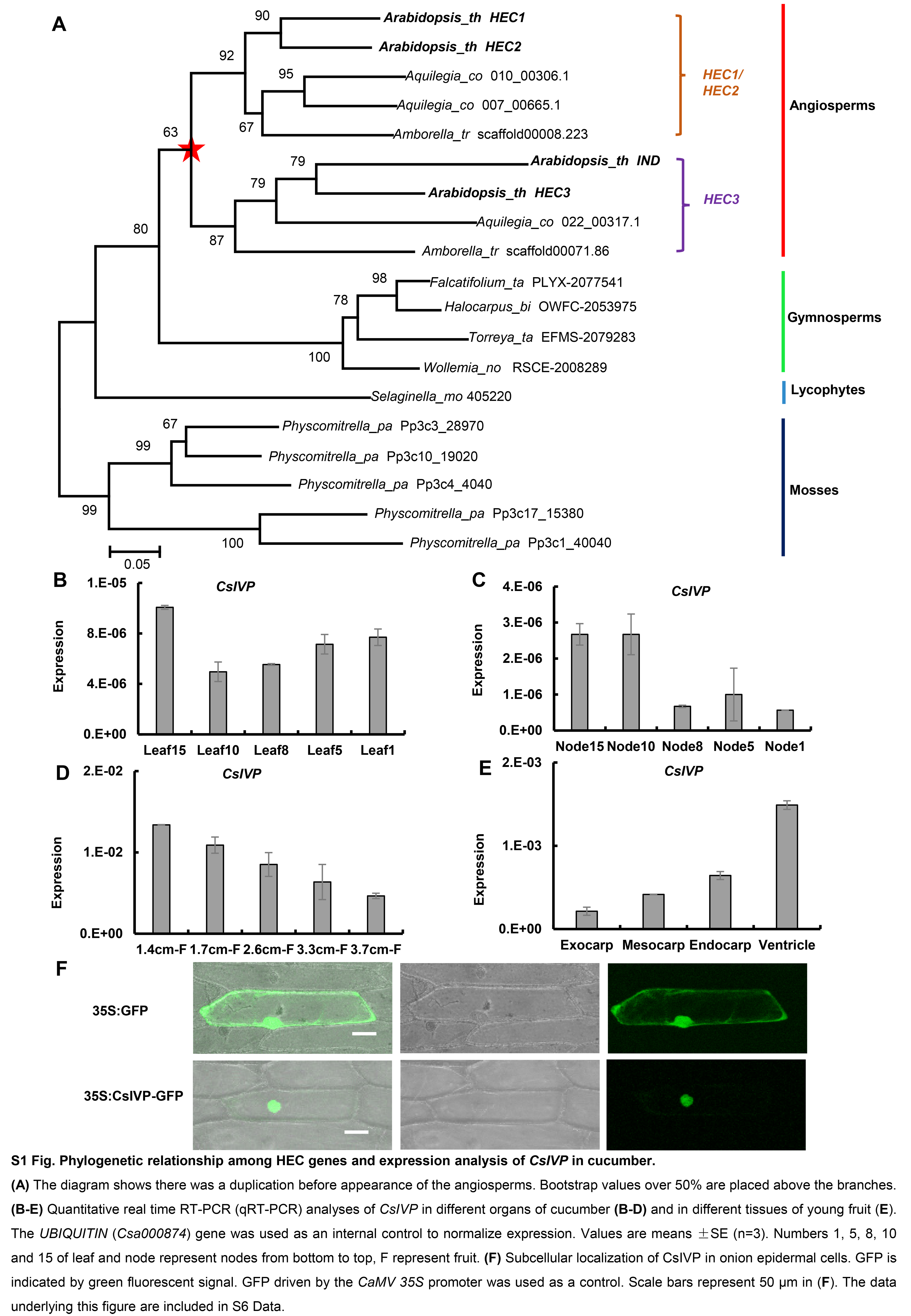

Supplement: S1 Fig — (A) The diagram shows there was a duplication before appearance of the angiosperms. Bootstrap values over 50% are placed above the branches. (B–E) qRT-PCR analyses of CsIVP in different organs of cucumber (panels B–D) and in different tissues of young fruit (panel E). The UBIQUITIN (Csa000874) gene was used as an internal control to normalize expression. Values are means ± SE (n = 3). Numbers 1, 5, 8, 10, and 15 of leaf and node represent nodes from bottom to top, and “F” represent fruit. (F) Subcellular localization of CsIVP in onion epidermal cells. GFP is indicated by green fluorescent signal. GFP driven by the CaMV 35S promoter was used as a control. Scale bars represent 50 μm in panel F. The data underlying this figure are included in S6 Data. (TIF) [file pbio.3000671.s001.tif]

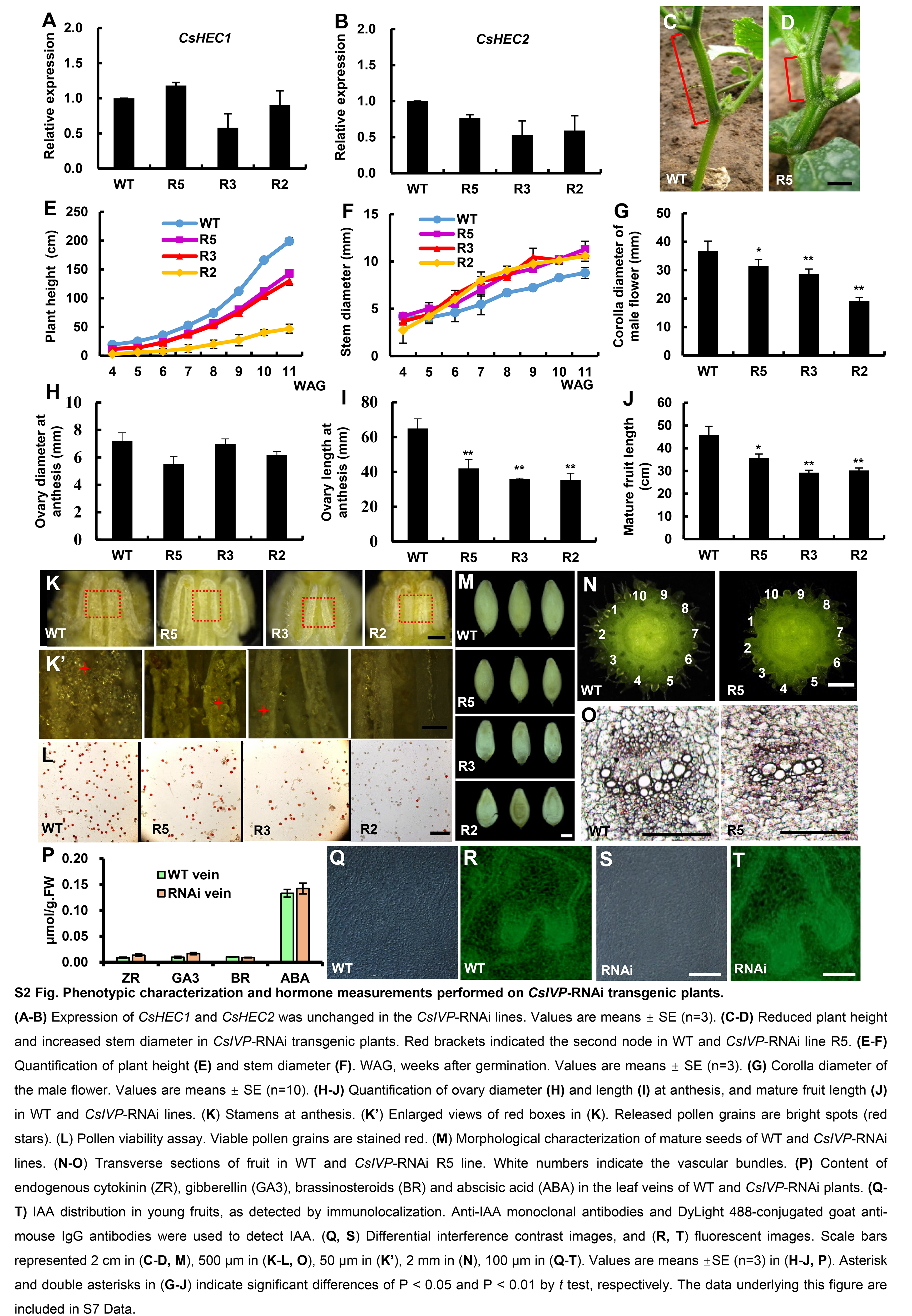

Supplement: S2 Fig — (A–B) Expression of CsHEC1 and CsHEC2 was unchanged in the CsIVP-RNAi lines. Values are means ± SE (n = 3). (C–D) Reduced plant height and increased stem diameter in CsIVP-RNAi transgenic plants. Red brackets indicated the second node in WT and CsIVP-RNAi line R5. (E–F) Quantification of plant height (panel E) and stem diameter (panel F). WAG, weeks after germination. Values are means ± SE (n = 3). (G) Corolla diameter of the male flower. Values are means ± SE (n = 10). (H-J) Quantification of ovary diameter (panel H) and length (panel I) at anthesis, and mature fruit length (panel J) in WT and CsIVP-RNAi lines. (K) Stamens at anthesis. (K’) Enlarged views of red boxes in panel K. Released pollen grains are bright spots (red stars). (L) Pollen viability assay. Viable pollen grains are stained red. (M) Morphological characterization of mature seeds of WT and CsIVP-RNAi lines. (N–O) Transverse sections of fruit in WT and CsIVP-RNAi R5 line. White numbers indicate the vascular bundles. (P) Content of ZR, GA3, BRs, and ABA in the leaf veins of WT and CsIVP-RNAi plants. (Q–T) IAA distribution in young fruits, as detected by immunolocalization. Anti-IAA monoclonal antibodies and DyLight 488–conjugated goat anti-mouse IgG antibodies were used to detect IAA. (Q, S) Differential interference contrast images, and (R, T) fluorescent images. Scale bars represented 2 cm in panels C–D and M; 500 μm in panels K–L and O; 50 μm in panel K’; 2 mm in panel N; and 100 μm in panels Q–T. Values are means ± SE (n = 3) in panels H–J and P. Asterisk and double asterisks in panels G–J indicate significant differences of P < 0.05 and P < 0.01 by t test, respectively. The data underlying this figure are included in S7 Data. ABA, abscisic acid; BR, brassinosteroid; GA3, gibberellic acid3; ZR, zeatin riboside (TIF) [file pbio.3000671.s002.tif]

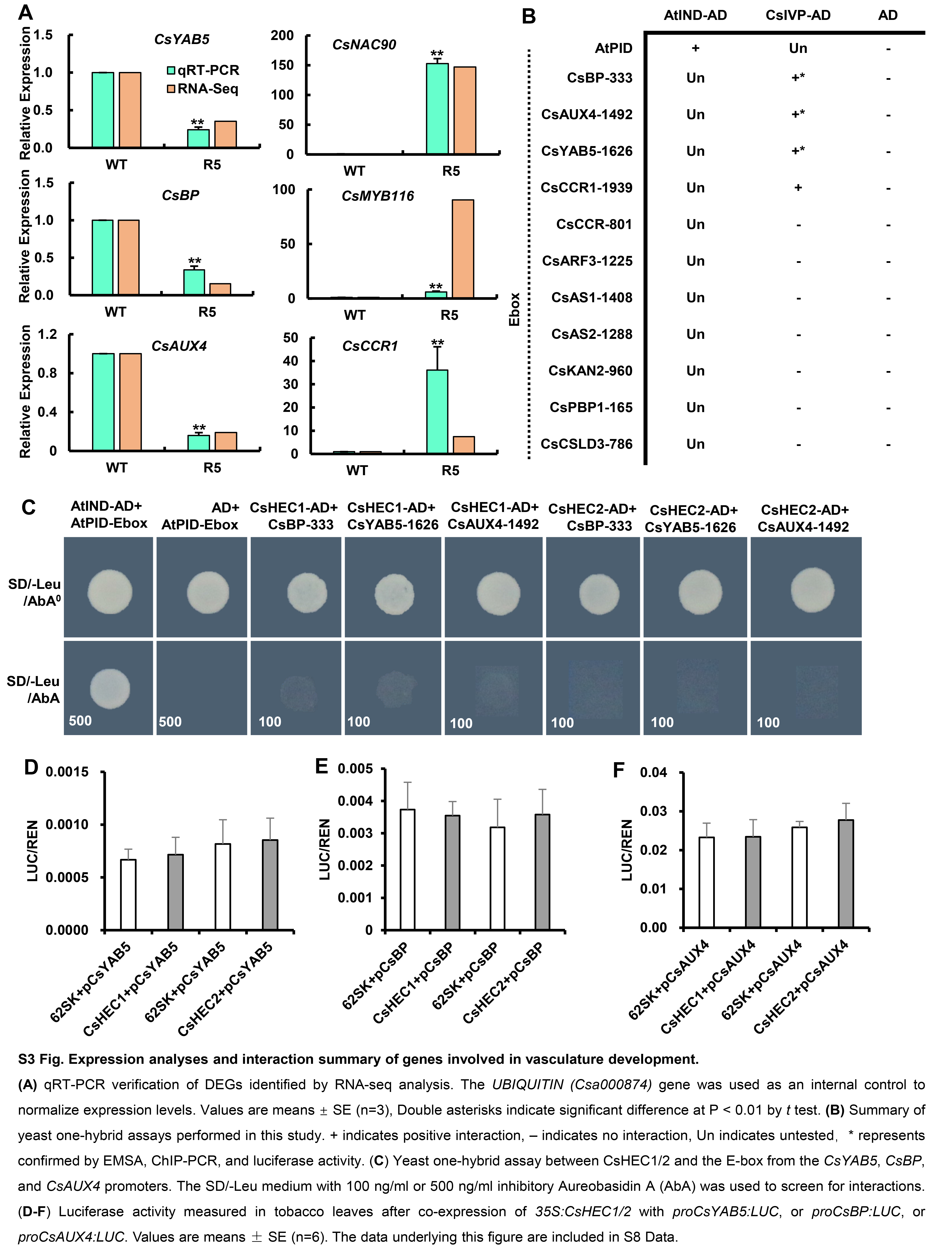

Supplement: S3 Fig — (A) qRT-PCR verification of DEGs identified by RNA-seq analysis. The UBIQUITIN (Csa000874) gene was used as an internal control to normalize expression levels. Values are means ± SE (n = 3), double asterisks indicate significant difference at P < 0.01 by t test. (B) Summary of yeast one-hybrid assays performed in this study. “+” indicates positive interaction, “−” indicates no interaction, “Un” indicates untested, “*” represents confirmed by EMSA, ChIP-PCR, and luciferase activity. (C) Yeast one-hybrid assay between CsHEC1/2 and the E-box from the CsYAB5, CsBP, and CsAUX4 promoters. The SD/-Leu medium with 100 ng/ml or 500 ng/ml inhibitory AbA was used to screen for interactions. (D–F) Luciferase activity measured in tobacco leaves after co-expression of 35S:CsHEC1/2 with proCsYAB5:LUC, or proCsBP:LUC, or proCsAUX4:LUC. Values are means ± SE (n = 6). The data underlying this figure are included in S8 Data. AbA, Aureobasidin A (TIF) [file pbio.3000671.s003.tif]

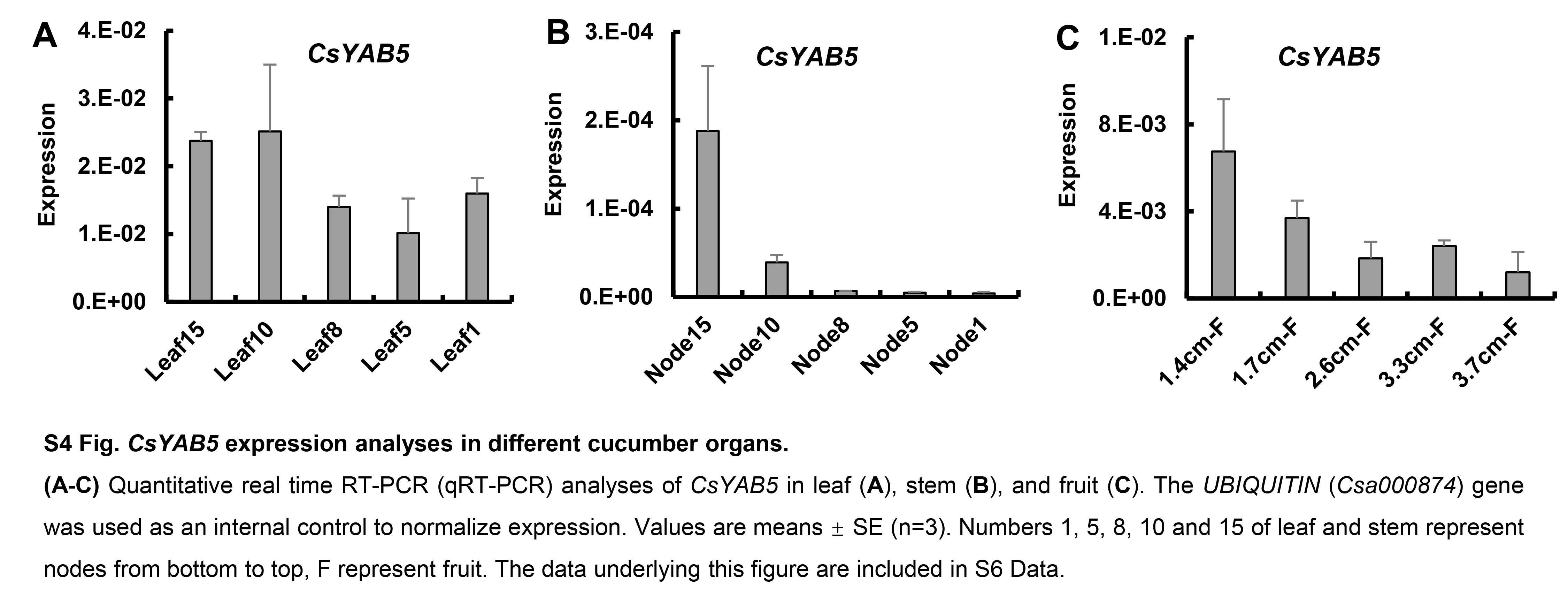

Supplement: S4 Fig — (A–C) qRT-PCR analyses of CsYAB5 in leaf (panel A), stem (panel B), and fruit (panel C). The UBIQUITIN (Csa000874) gene was used as an internal control to normalize expression. Values are means ± SE (n = 3). Numbers 1, 5, 8, 10, and 15 of leaf and stem represent nodes from bottom to top; “F” represent fruit. The data underlying this figure are included in S6 Data. (TIF) [file pbio.3000671.s004.tif]

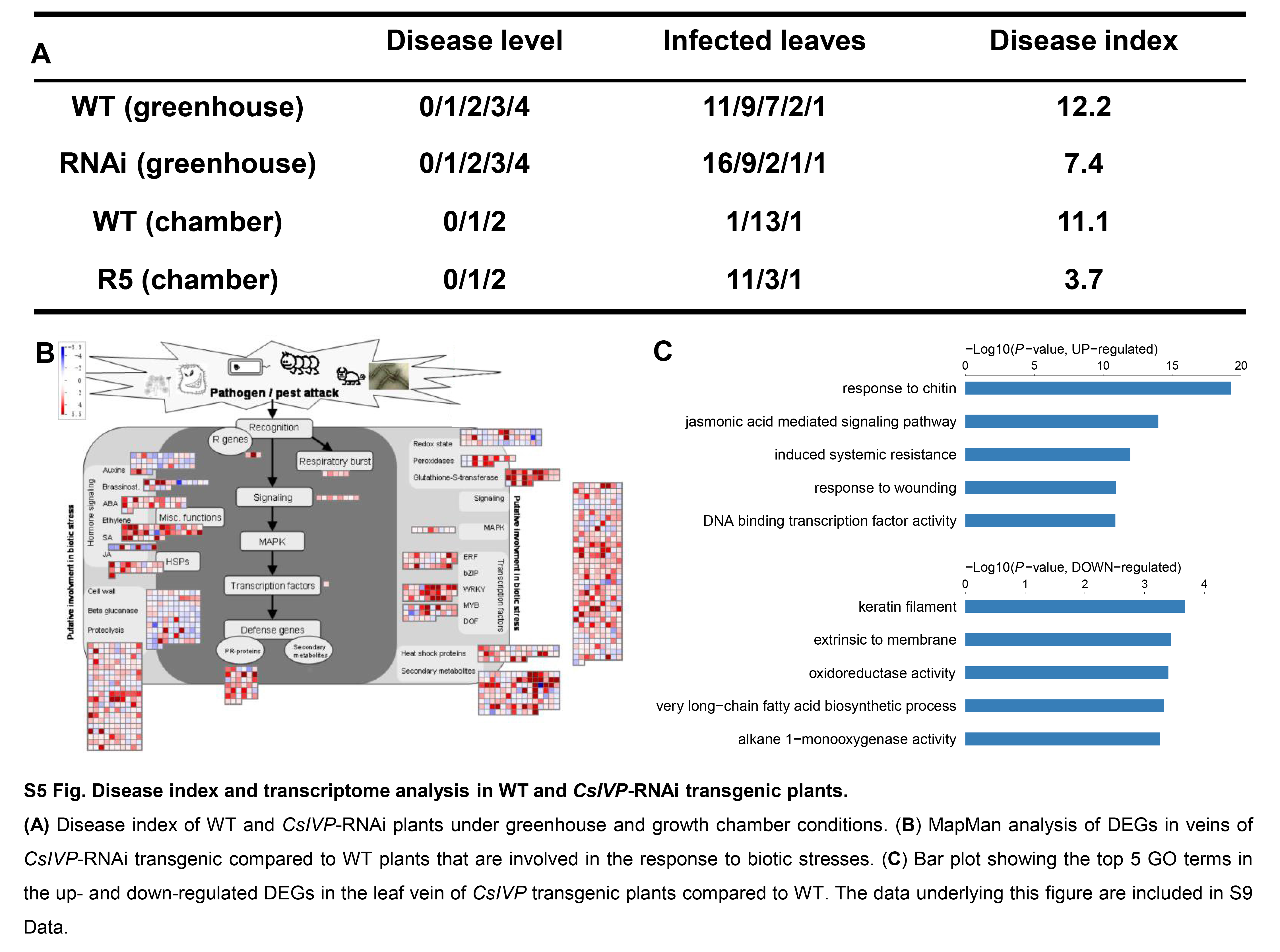

Supplement: S5 Fig — (A) Disease index of WT and CsIVP-RNAi plants under greenhouse and growth chamber conditions. (B) MapMan analysis of DEGs in veins of CsIVP-RNAi transgenic compared to WT plants that are involved in the response to biotic stresses. (C) Bar plot showing the top 5 GO terms in the up- and down-regulated DEGs in the leaf vein of CsIVP transgenic plants compared to WT. The data underlying this figure are included in S9 Data. (TIF) [file pbio.3000671.s005.tif]

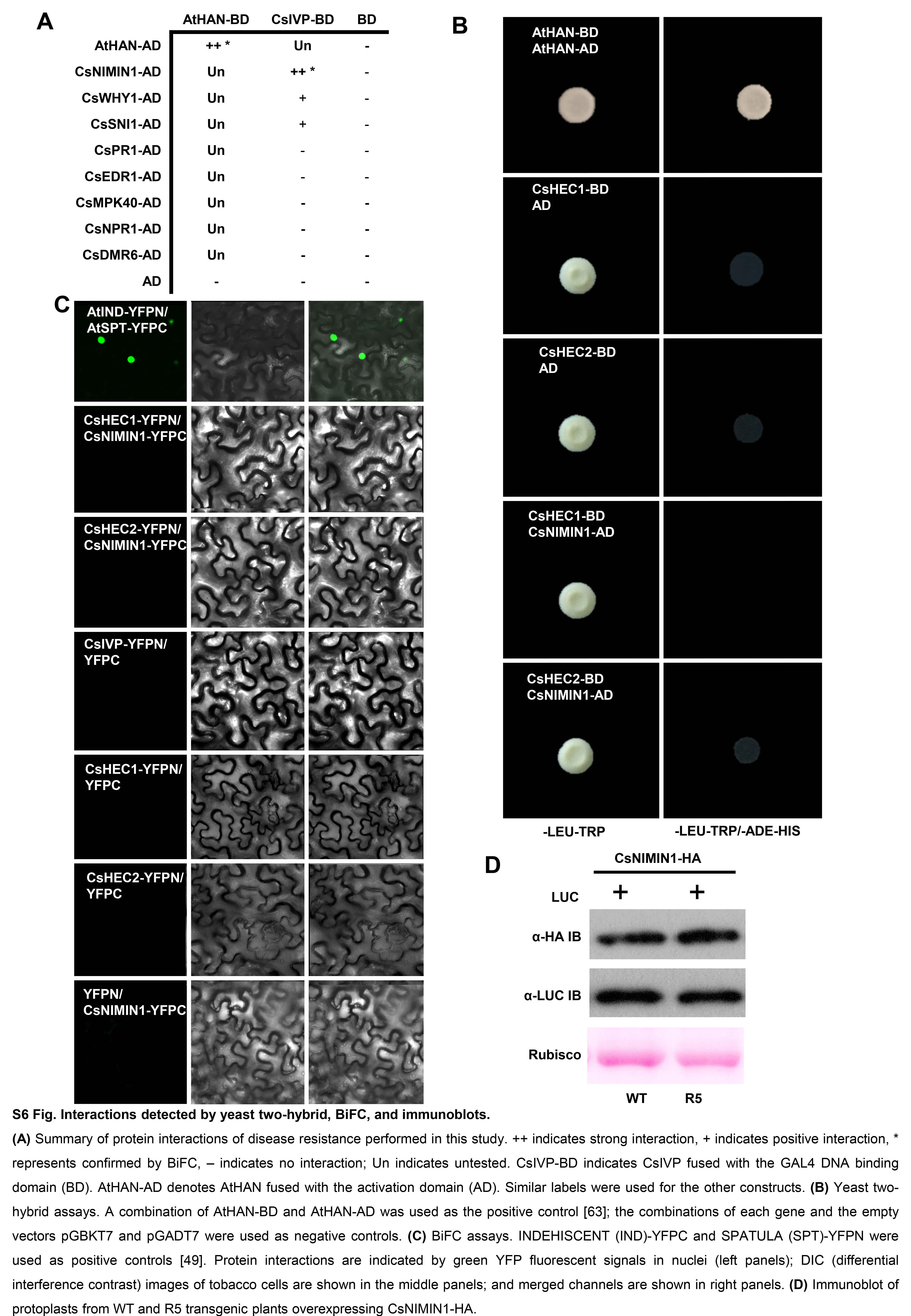

Supplement: S6 Fig — (A) Summary of protein interactions of disease resistance performed in this study. “++” indicates strong interaction, “+” indicates positive interaction, “*” represents confirmed by BiFC, “–” indicates no interaction; “Un” indicates untested. CsIVP-BD indicates CsIVP fused with the GAL4 DNA binding domain. AtHAN-AD denotes AtHAN fused with the activation domain. Similar labels were used for the other constructs. (B) Y2H assays. A combination of AtHAN-BD and AtHAN-AD was used as the positive control [63]; the combinations of each gene and the empty vectors pGBKT7 and pGADT7 were used as negative controls. (C) BiFC assays. IND-YFPC and SPT-YFPN were used as positive controls [49]. Protein interactions are indicated by green YFP fluorescent signals in nuclei (left panels); DIC images of tobacco cells are shown in the middle panels; and merged channels are shown in right panels. (D) Immunoblot of protoplasts from WT and R5 transgenic plants overexpressing CsNIMIN1-HA. AD, activation domain; BD, binding domain; DIC, differential interference contrast; IND, INDEHISCENT; SPT, SPATULA; Y2H, yeast two-hybrid (TIF) [file pbio.3000671.s006.tif]
