# Supplementary material for: CsIVP functions in vasculature development and downy mildew resistance in cucumber
Source: PLoS Biol. 2020 Mar 23;18(3):e3000671. doi: 10.1371/journal.pbio.3000671 (PMC7117775; doi:10.1371/journal.pbio.3000671)
Supplement: S1 Table — (DOCX) [file pbio.3000671.s007.docx]

| **S1 Table. Summary of transcriptome sequencing data** | | | | | |  |
| --- | --- | --- | --- | --- | --- | --- |
| **Sample** | **Raw Reads** | **Clean Reads** | **Mapped** | **Uniquely_mapped** |  | |
| WT vein rep1 | 21.58 | 20.13 (93.3%) | 17.98 (83.32%) | 17.68 (81.93%) |  | |
| WT vein rep2 | 24.92 | 23.25 (93.3%) | 20.56 (82.50%) | 20.21 (81.10%) |  | |
| R5 vein rep1 | 22.59 | 20.93 (92.7%) | 18.5 (81.89%) | 18.21 (80.61%) |  | |
| R5 vein rep2 | 22.86 | 21.34 (93.3%) | 19.01 (83.16%) | 18.73 (81.93%) |  | |
| WT fruit rep1 | 26.38 | 25.01 (94.8%) | 22.65 (85.86%) | 22.3 (84.53%) |  | |
| WT fruit rep2 | 27.13 | 26.10 (96.2%) | 23.53 (86.73%) | 23.16 (85.37%) |  | |
| R5 fruit rep1 | 22.64 | 21.02 (92.8%) | 18.37 (81.14%) | 18.06 (79.77%) |  | |
| R5 fruit rep2 | 26.27 | 24.89 (94.7%) | 21.91 (83.40%) | 21.59 (82.19%) |  | |

Rep1 and rep2 indicated two biological replicates.
