# Supplementary material for: CsIVP functions in vasculature development and downy mildew resistance in cucumber
Source: PLoS Biol. 2020 Mar 23;18(3):e3000671. doi: 10.1371/journal.pbio.3000671 (PMC7117775; doi:10.1371/journal.pbio.3000671)
Supplement: S3 Table — (DOCX) [file pbio.3000671.s009.docx]

**S3 Table. Examples of genes involved in vascular development that are differentially expressed in the veins of R5 vs WT in cucumber**

| **Gene ID** | **Gene name** | **LogFC** | **p-value** |
| --- | --- | --- | --- |
| Csa2G352430 | BP (KNOTTED1-LIKE HOMEOBOX GENE 1) | -2.7 | 4.1E-05 |
| Csa2G006820 | YAB5 (YABBY family protein 5) | -1.5 | 7.5E-08 |
| Csa5G160210 | FIL (YABBY family protein 1) | -3.0 | 2.8E-15 |
| Csa2G034560 | KNAT2 (KNOTTED1-LIKE HOMEOBOX GENE 2) | -2.2 | 9.7E-03 |
| Csa5G600390 | KNAT6 (KNOTTED1-LIKE HOMEOBOX GENE 6) | 1.8 | 1.1E-06 |
| Csa6G425720 | NAC domain containing protein 90 | 7.1 | 3.4E-11 |
| Csa4G011770 | NAC domain containing protein 83 | 2.2 | 8.5E-13 |
| Csa6G092010 | NAC domain containing protein 83 | 1.3 | 5.7E-07 |
| Csa1G042350 | MYB116 (myb domain protein 116) | 6.5 | 2.1E-39 |
| Csa1G024160 | MYB116b (myb domain protein 116) | 5.8 | 7.8E-18 |
| Csa6G362930 | ACL5 (ACAULIS 5) | 1.3 | 3.6E-03 |
| Csa6G135460 | APL (ALTERED PHLOEM DEVELOPMENT) | 1.5 | 2.8E-06 |
| Csa1G015700 | IRX6 (IRREGULAR XYLEM 6) | -1.0 | 9.0E-03 |
| Csa4G129050 | COBRA-like protein family | 2.6 | 1.5E-13 |
| Csa3G717370 | CCR1 (cinnamoyl coa reductase 1) | 5.4 | 6.0E-12 |
| Csa3G716870 | CCR (cinnamoyl coa reductase) | 5.5 | 5.3E-09 |
| Csa6G091870 | CCR-like | -1.9 | 7.2E-10 |
| Csa3G598910 | CCR-like | 4.0 | 2.1E-04 |
| Csa2G382670 | Eukaryotic elongation factor 5A-1 | -1.1 | 6.4E-03 |
| Csa4G002500 | Cellulose synthase-like D5 | -1.8 | 5.1E-07 |
| Csa7G041990 | Cellulose synthase-like B1 | -1.3 | 2.0E-04 |
| Csa2G416190 | Cellulose synthase-like B3 | 3.4 | 5.7E-28 |
| Csa2G416170 | Cellulose synthase-like B3 | 2.8 | 3.0E-14 |
| Csa2G433910 | Cellulose synthase-like D3 | 3.6 | 2.4E-08 |
| Csa3G180430 | ANL2 (ANTHOCYANINLESS 2) | 3.1 | 9.3E-11 |
| Csa4G046770 | Laccase 11 | 1.5 | 1.8E-03 |
| Csa3G734120 | Laccase 17 | -1.6 | 1.4E-03 |
| Csa1G533470 | Laccase 3 | -3.0 | 1.5E-08 |
| Csa7G429550 | Callose synthase 1 | 2.3 | 2.5E-10 |
| Csa2G302250 | Callose synthase 5 | 1.4 | 5.3E-05 |
| Csa1G013210 | plasmodesmata callose-binding protein 3 | 9.0 | 3.0E-36 |
| Csa5G606660 | plasmodesmata callose-binding protein 3 | 3.9 | 7.9E-10 |
| Csa5G173520 | Phloem protein 2-A14 | 2.4 | 1.4E-06 |
| Csa1G708660 | Phloem protein 2-A9 | 1.1 | 2.7E-04 |
| Csa3G132020 | Phloem protein 2-B12 | 2.8 | 2.6E-08 |
| Csa2G032230 | Phloem protein 2-B15 | 2.3 | 1.0E-04 |
| Csa1G002870 | SIEVE-ELEMENT-OCCLUSION-RELATED 1 | 1.4 | 1.3E-04 |
| Csa2G193340 | SIEVE-ELEMENT-OCCLUSION-RELATED 1 | -2.4 | 1.2E-16 |
