# Supplementary material for: CsIVP functions in vasculature development and downy mildew resistance in cucumber
Source: PLoS Biol. 2020 Mar 23;18(3):e3000671. doi: 10.1371/journal.pbio.3000671 (PMC7117775; doi:10.1371/journal.pbio.3000671)
Supplement: S4 Table — (DOCX) [file pbio.3000671.s010.docx]

**S4 Table. Examples of auxin-related genes differentially expressed in the veins of**

**R5 vs WT in cucumber**

| **Gene ID** | **Gene name** | **LogFC** | **p-value** |
| --- | --- | --- | --- |
| Csa1G071800 | pinoid-binding protein 1 | 10.4 | 7.1E-25 |
| Csa7G041870 | Auxin-responsive family protein | 6.7 | 5.8E-17 |
| Csa7G033410 | Auxin-responsive family protein | 3.4 | 3.1E-19 |
| Csa4G007100 | Auxin-responsive GH3 family protein | 2.5 | 1.7E-15 |
| Csa7G448680 | SAUR-like auxin-responsive protein family | 2.2 | 1.5E-06 |
| Csa3G198490 | Auxin-responsive GH3 family protein | 2.1 | 2.6E-04 |
| Csa3G000170 | Auxin efflux carrier family protein | 1.8 | 7.4E-06 |
| Csa1G051690 | SAUR-like auxin-responsive protein family | 1.7 | 3.6E-03 |
| Csa6G137590 | SAUR-like auxin-responsive protein family | 1.6 | 2.7E-05 |
| Csa3G119760 | Auxin-responsive GH3 family protein | 1.5 | 3.3E-05 |
| Csa1G569400 | Dormancy/auxin associated family protein | 1.5 | 1.6E-04 |
| Csa7G009150 | SAUR-like auxin-responsive protein family | 1.5 | 1.3E-03 |
| Csa6G092560 | SAUR-like auxin-responsive protein family | 1.3 | 1.1E-03 |
| Csa3G431430 | Auxin-responsive GH3 family protein | 1.0 | 9.1E-03 |
| Csa3G883020 | SAUR-like auxin-responsive protein family | -3.2 | 1.7E-03 |
| Csa2G011420 | AUX/IAA transcriptional regulator family protein | -2.4 | 1.4E-09 |
| Csa7G018810 | SAUR-like auxin-responsive protein family | -2.4 | 3.8E-03 |
| Csa7G010800 | like AUXIN RESISTANT 2 | -2.3 | 1.8E-10 |
| Csa1G427480 | Auxin efflux carrier family protein | -2.2 | 8.9E-03 |
| Csa2G258100 | SAUR-like auxin-responsive protein family | -2.1 | 1.0E-09 |
| Csa2G258750 | SAUR-like auxin-responsive protein family | -2.0 | 4.0E-04 |
| Csa3G134550 | auxin-induced protein 13 | -1.7 | 3.9E-08 |
| Csa3G646510 | SAUR-like auxin-responsive protein family | -1.7 | 2.1E-04 |
| Csa3G143580 | AUX/IAA transcriptional regulator family protein | -1.7 | 1.1E-05 |
| Csa7G008430 | SAUR-like auxin-responsive protein family | -1.6 | 1.7E-03 |
| Csa6G106780 | SAUR-like auxin-responsive protein family | -1.6 | 5.6E-04 |
| Csa7G009020 | SAUR-like auxin-responsive protein family | -1.6 | 3.7E-03 |
| Csa2G170820 | auxin-induced protein 13 | -1.5 | 5.0E-07 |
| Csa2G258780 | SAUR-like auxin-responsive protein family | -1.4 | 1.7E-03 |
| Csa7G009040 | SAUR-like auxin-responsive protein family | -1.3 | 1.2E-02 |
| Csa7G378530 | AUX/IAA transcriptional regulator family protein | -1.2 | 6.0E-04 |
