# Supplementary material for: CsIVP functions in vasculature development and downy mildew resistance in cucumber
Source: PLoS Biol. 2020 Mar 23;18(3):e3000671. doi: 10.1371/journal.pbio.3000671 (PMC7117775; doi:10.1371/journal.pbio.3000671)
Supplement: S5 Table — (DOCX) [file pbio.3000671.s011.docx]

| **E-box** | **CsIVP-AD** |
| --- | --- |
| CsYAB5-513 | - |
| CsYAB5-616 | - |
| CsYAB5-1626 | +* |
| CsYAB5+382-intron | Un |
| CsYAB5+890-intron | Un |
| CsYAB5+1216-intron | Un |
| CsYAB5+2025-intron | Un |
| CsYAB5+2150-intron | Un |
| CsBP-333 | +* |
| CsBP-1212 | - |
| CsBP-2692 | - |
| CsBP+1779- intron | Un |
| CsBP+2244- intron | Un |
| CsBP+2805- intron | Un |
| CsBP+2931- intron | Un |
| CsAUX4-212 | - |
| CsAUX4-1342 | - |
| CsAUX4-1492 | +* |
| CCR1-925 | - |
| CCR1-1367 | - |
| CCR1-1939 | + |
| CCR1+642-intron | Un |

**S5 Table. Summary of CsIVP binding to the E-box in promoters and introns of *CsYAB5, CsBP, CsAUX4,* and *CsCCR1* genes**

+ indicates positive interaction, – indicates no interaction, Un indicates untested，* represents confirmed by EMSA, ChIP-PCR and Luciferase activity.
