# Supplementary material for: CsIVP functions in vasculature development and downy mildew resistance in cucumber
Source: PLoS Biol. 2020 Mar 23;18(3):e3000671. doi: 10.1371/journal.pbio.3000671 (PMC7117775; doi:10.1371/journal.pbio.3000671)
Supplement: S6 Table — (DOCX) [file pbio.3000671.s012.docx]

| **S6 Table. Primers used in this study** | |
| --- | --- |
| **Primers for gene amplification and vector construction** | |
| *CsIVP-clone-F* | ATGGACCCCCATCACCTCTCAA |
| *CsIVP-clone-R* | TCACCATCCTGTTGGTGTAATAGCA |
| *CsIVP-Sense-F* | GGACTAGTCCATCACCTCTCAAACCCTCC |
| *CsIVP-Sense-R* | CGGGATCCCGATACATCATCTCCTTCATCGC |
| *CsIVP-Antisense-F* | TTGGCGCGCCAAATCACCTCTCAAACCCTCC |
| *CsIVP-Antisense-R* | GCGATTTAAATCGGATACATCATCTCCTTCATCGC |
| *CsIVP-GFP-F* | TGCTCTAGAGCAATGGACCCCCATCACCT |
| *CsIVP-GFP-R*  *CsYAB5-Sense-F*  *CsYAB5-Sense-R*  *CsYAB5-Antisense-F*  *CsYAB5-Antisense-R* | TCCCCCGGGGGACCATCCTGTTGGTGTAATAGC  CTTTGATGGGGATCCGTTGCTTTGATCCTTTGGATTTCC  ACTCTAGGGACTAGTCAATCTCTCTCATGGCAAAATTC  ACCATGGGGCGCGCCCAATCTCTCTCATGGCAAAATTC  TCTGGGGATTTAAATGTTGCTTTGATCCTTTGGATTTCC |
| **Primers for qRT-PCR** | |
| *CsIVP-Q-F* | CCATTCGGAAGCCTAAGC |
| *CsIVP-Q-R* | GCGGTTGACTTGACTGTAG |
| *CsHEC1-Q-F* | ACAAATGGAAAAAATCCCTGAA |
| *CsHEC1-Q-R* | TGTTGATGAATAATGGAGGCGA |
| *CsHEC2-Q-F* | ATTTCTCCGACTACTCCCCGC |
| *CsHEC2-Q-R* | CCATTCCTCCCCCACTTACG |
| *CsAUX4-Q-F* | TCTCCCATCAACCGAACC |
| *CsAUX4-Q-R* | GGAGGGGTATTTTCTTGGCT |
| *CsCCR1-Q-F* | GTTTTCCACACCGCCTCTC |
| *CsCCR1-Q-R* | TCCAACAAGACTCGTCCACC |
| *CsMYB116-Q-F* | TCCCAGACTTCGTTTGGTTTT |
| *CsMYB116-Q-R* | TCAGCATCTCTCCTCCGCC |
| *CsNAC90-Q-F* | GCGACAGGGTCTCCAACTTAC |
| *CsNAC90-Q-R* | GGGAATGAGGAGGAATGATGTC |
| *CsBP-Q-F* | ATTCTTCATCTCCTGTGGGCA |
| *CsBP-Q-R* | CAGTAGTAGTGGTAGTGGGAGGCT |
| *CsYAB5-Q-F* | TTCTCAGGCATCAAACCATAGC |
| *CsYAB5-Q-R* | CTTTGGATTTCCTCTGTCGGT |
| *CsNPR1-Q-F* | CTCCAAGTTTTGCCTCGTCG |
| *CsNPR1-Q-R* | TCTGAAAGCCACCAACTTAGACAT |
| *CsPR-1-F* | GATACCAATGAATGTGCCGAC |
| *CsPR-1-R*  *PsITS-Q-F*  *PsITS-Q-R* | CAAATCGGTTTCTCATTCACG  CGGGGGTTTTGTTTGGCGGT  CGAAGCCCAAACGCTCGCCA |
| *CsUBIeq-Q-F* | CACCAAGCCCAAGAAGATC |
| *CsUBI-eq-Q-R* | TAAACCTAATCACCACCAGC |
| **Primers for in situ probes** | |
| *CsIVP-T7*  TGTAATACGACTCACTATAGGG TTCTTCCGGCTCTTCTTC | |
| *CsIVP-Sp6* GATTTAGGTGACACTATAGAATGCT ATCACCTCTCAAACCCTCC  *CsYAB5-T7* TGTAATACGACTCACTATAGGGGTCGGTTAATGAGCCTTTG  *CsYAB5-SP6* GATTTAGGTGACACTATAGAATGCTATGTCAAGCTGCATCTCTAG | |
| **Primers for yeast two-hybrid and BiFC** | |
| *AtHAN-YTH-F* | GGAATTCCATATGATGATGCAGACTCCGTACACT |
| *AtHAN -YTH-R* | CGGGATCCCTCTGGTAAAGTCATGGACAAGAC |
| *CsIVP-YTH-F* | GGAATTCCATATGATGGACCCCCATCACCTC |
| *CsIVP-YTH-R* | CGGGATCCCTCACCATCCTGTTGGTGTAATAG |
| *CsHEC1-YTH-F* | GGGAATTCCATATGATGGAAAATGATGATTTAAAATCGG |
| *CsHEC1-YTH-R* | CGCGGATCCTCAAGGTTGGGATTGATGATGAT |
| *CsHEC2-YTH-F* | GGGAATTCCATATGATGGACGATATCGACATCCTCAAA |
| *CsHEC2-YTH-R* | CGCGGATCCTCAAGACTGCATTTGCAAAGAAGC |
| *CsNIMIN1-YTH-F* | GGAATTCCATATGATGGAAAGTGGAAGAAAAAGAAGAAA |
| *CsNIMIN1-YTH-R* | CGGGATCCTCAAAGAGAGAGATTAAGGTCAAGCC |
| *AtIND-BiFC-F* | TGCTCTAGAATGGAAAATGGTATGTATAAAAAG |
| *AtIND-BiFC-R* | CGCGGATCCGGGTTGGGAGTTGTGGTA |
| *AtSPT-BiFC-F* | TGCTCTAGAATGATATCACAGAGAGAAGAAAGA |
| *AtSPT-BiFC-R* | CGCGGATCCAGTAATTCGATCTTTTAGGTCAG |
| *CsIVP-BiFC-F* | TGCTCTAGA ATGGACCCCCATCACCTC |
| *CsIVP-BiFC-R* | CGCGGATCC CCATCCTGTTGGTGTAATAGC |
| *CsHEC1-BiFC-F* | GCTCTAGAATGGAAAATGATGATTTAAAATCGG |
| *CsHEC1-BiFC-R* | TCCCCCCGGGAGGTTGGGATTGATGATGAT |
| *CsHEC2-BiFC-F* | GCTCTAGAATGGACGATATCGACATCCTCAAA |
| *CsHEC2-BiFC-R* | TCCCCCCGGGAGACTGCATTTGCAAAGAAGC |
| *CsNIMIN1-BiFC-F* | CGCGCCACTAGTGGATCCATGGAAAGTGGAAGAAAAAGAAGAAA |
| *CsNIMIN1-BiFC-R* | AGTACTATCGATGGATCCAAGAGAGAGATTAAGGTCAAGCC |
| **Primers for yeast one-hybrid, LUC activity measure assay** | |
| *AtPID-Ebox-F* | AGCTTTCTCACGCGTTGTCTCACGCGTTGTCTCACGCGTTGG |
| *AtPID-Ebox-R* | TCGACCAACGCGTGAGACAACGCGTGAGACAACGCGTGAGAA |
| *CsCCR1-1939-F* | AGCTTTTCCACTTGTGCTTCCACTTGTGCTTCCACTTGTGCG |
| *CsCCR1-1939-R* | TCGACGCACAAGTGGAAGCACAAGTGGAAGCACAAGTGGAA |
| *CsAUX4-1492-F* | AGCTTGAGCACATGGGAGAGCACATGGGAGAGCACATGGGAG |
| *CsAUX4-1492-R* | TCGACTCCCATGTGCTCTCCCATGTGCTCTCCCATGTGCTCA |
| *CsYAB5-1626-F* | AGCTTAACCAATTGAGTAACCAATTGAGTAACCAATTGAGTG |
| *CsYAB5-1626-R* | TCGACACTCAATTGGTTACTCAATTGGTTACTCAATTGGTTA |
| *CsBP-333-F* | AGCTTCCTCAACTGAGACCTCAACTGAGACCTCAACTGAGAG |
| *CsBP-333-R* | TCGACTCTCAGTTGAGGTCTCAGTTGAGGTCTCAGTTGAGGA |
| *CsAS1-1408-F* | AGCTTTATCAAATGAAATATCAAATGAAATATCAAATGAAAG |
| *CsAS1-1408-R*  *CsIVP-62SK-F*  *CsIVP-62SK-R*  *CsHEC1-62SK-F* | TCGACTTTCATTTGATATTTCATTTGATATTTCATTTGATAA  AGAACTAGTGGATCCATGGACCCCCATCACCTC  GGTATCGATAAGCTTTCACCATCCTGTTGGTGTAATAGC  AGAACTAGTGGATCCATGGAAAATGATGATTTAAAATC |
| *CsHEC1-62SK-R* | GGTATCGATAAGCTTTCAAGGTTGGGATTGATG |
| *CsHEC2-62SK-F* | AGAACTAGTGGATCCATGGACGATATCGACATC |
| *CsHEC2-62SK-R* | GGTATCGATAAGCTTTCAAGACTGCATTTGCA |
| *proCsYAB5-0800F*  *proCsYAB5-0800R*  *ProCsAUX4-0800F*  *ProCsAUX4-0800R*  *ProCsBP-0800F*  *ProCsBP-0800R* | GGTATCGATAAGCTTGCAACAAATTGAGGGGTG  AGAACTAGTGGATCCGTTTGAAAATTAAAAATAACAAAGCTTTTTCA  GGTATCGATAAGCTTATGTGTTGACATGTCGACATG  AGAACTAGTGGATCCTGTTGAATTTTTGCTTCTCTG  GGTATCGATAAGCTTCCTGAGTTTGGACTAGTCTGTCC  AGAACTAGTGGATCCTACAAAATTAAATAAAAAACATATAATAAT |
| **Primers for EMSA, CHIP** | |
| *CsBP-F* | ACTTTAATGGCGCACCCTCAACTGAGATATCTATACCC |
| *CsBP-R* | GGGTATAGATATCTCAGTTGAGGGTGCGCCATTAAAGT |
| *CsBP-F-Biotin* | Biotin-ACTTTAATGGCGCACCCTCAACTGAGATATCTATACCC |
| *CsCCR1-F* | CAAAATTGACTTCTTCCACTTGTGCAGTCAAACAGTTC |
| *CsCCR1-R* | GAACTGTTTGACTGCACAAGTGGAAGAAGTCAATTTTG |
| *CsCCR1-F-BIotin* | Biotin-CAAAATTGACTTCTTCCACTTGTGCAGTCAAACAGTTC |
| *CsAUX4-F* | AGGGATGGAGGTGAGCACATGGGAGGGCCGCTGCC |
| *CsAUX4-R* | GGCAGCGGCCCTCCCATGTGCTCACCTCCATCCCT |
| *CsAUX4-F-Biotin* | Biotin-AGGGATGGAGGTGAGCACATGGGAGGGCCGCTGCC |
| *CsYAB5-F* | TATAAAATTGTTTTAACCAATTGAGTTATATACATTCAA |
| *CsYAB5-R* | TTGAATGTATATAACTCAATTGGTTAAAACAATTTTATA |
| *CsYAB5F-Biotin*  *CsBP-333ChipF*  *CsBP-333ChipR*  *CsYAB5-1626ChipF*  *CsYAB5-1626ChipR*  *CsAUX4-1492ChipF*  *CsAUX4-1492ChipR*  *CsCCR1-1939-ChipF* | Biotin-TATAAAATTGTTTTAACCAATTGAGTTATATACATTCAA  CGTGGTTGTCTCCTAGATG  GAAGGTGTGTTTTGGGTATAGA  GAGCAAAAATAAATCCCAAATTGTT  TGAGGATGTCGGTATCACTT  GTGATCATGTCGGACGAAGAA  AACTATTTGGCTATGGCGGTT  TACAGACAAACACACTTCAGATT |
| *CsCCR1-1939-ChipR* | GAGAGAACTGTTTGACTGCA |
| *Tubulin F*  *Tubulin R* | ACGCTGTTGGTGGTGGTAC  GAGAGGGGTAAACAGTGAATC |
|  | |
