# Supplementary material for: CsIVP functions in vasculature development and downy mildew resistance in cucumber
Source: PLoS Biol. 2020 Mar 23;18(3):e3000671. doi: 10.1371/journal.pbio.3000671 (PMC7117775; doi:10.1371/journal.pbio.3000671)
Supplement: S7 Table — (DOCX) [file pbio.3000671.s013.docx]

**S7 Table. Gene accession numbers used for phylogenetic analysis**

| **Name** | **Species** | **Family** | **Accession** |
| --- | --- | --- | --- |
| *Glycine_ma* 05G190600.1 | *Glycine max* | Fabaceae | Glyma.05G190600.1 |
| *Glycine_ma* 08G156000.1 | *Glycine max* | Fabaceae | Glyma.08G156000.1 |
| *Glycine_ma* 06G152300.1 | *Glycine max* | Fabaceae | Glyma.06G152300.1 |
| *Glycine_ma* 04G214100.1 | *Glycine max* | Fabaceae | Glyma.04G214100.1 |
| *Phaseolus_vu* 002G271000.1 | *Phaseolus vulgaris* | Fabaceae | Phvul_002G271000.1 |
| *Phaseolus_vu* 009G149200.1 | *Phaseolus vulgaris* | Fabaceae | Phvul_009G149200.1 |
| *Prunus_pe* 6G159200.1 | *Prunus persica* | Rosaceae | Prupe_6G159200.1 |
| *Malus_do* MDP0000711774 | *Malus domestica* | Rosaceae | MDP0000711774 |
| *Fragaria_ve* mrna17881.1 | *Fragaria vesca* | Rosaceae | Frave_mrna17881.1 |
| *Cucumis _sa CsIVP* | *Cucumis sativus* | Cucurbitaceae | Cucsa_101020.1 |
| *Cucumis_me* XP_016899193.1 | *Cucumis melo* | Cucurbitaceae | XP_016899193.1 |
| *Citrullus_vu*  Cla008442 | *Citrullus vulgaris Schrad* | Cucurbitaceae | Cla008442 |
| *Populus_tr* 007G108000.1 | *Populus trichocarpa* | Salicaceae | Potri_007G108000.1 |
| *Populus_tr* T107900.1 | *Populus trichocarpa* | Salicaceae | Potri_T107900.1 |
| *Populus_tr* 005G060900.1 | *Populus trichocarpa* | Salicaceae | Potri_005G060900.1 |
| *Salix_pu* 0550s0030.1 | *Salix purpurea* | Salicaceae | SapurV1A.0550s0030.1 |
| *Salix_pu* 0352s0020.1 | *Salix purpurea* | Salicaceae | SapurV1A.0352s0020.1 |
| *Ricinus_co* 30136.m001043 | *Ricinus communis* | Euphorbiaceae | Ricco_30136.m001043 |
| *Arabidopsis_th HEC1* | *Arabidopsis thaliana* | Brassicaceae | AT5G67060.1 |
| *Arabidopsis_th HEC2* | *Arabidopsis thaliana* | Brassicaceae | AT3G50330.1 |
| *Arabidopsis_th HEC3* | *Arabidopsis thaliana* | Brassicaceae | AT5G09750.1 |
| *Arabidopsis_th IND* | *Arabidopsis thaliana* | Brassicaceae | AT4G00120.1 |
| *Capsella_gr HEC3* | *Capsella grandiflora* | Brassicaceae | Cagra_1085s0086.1 |
| *Capsella_gr IND* | *Capsella grandiflora* | Brassicaceae | Cagra_1261s0004.1 |
| *Capsella_ru HEC3* | *Capsella rubella* | Brassicaceae | Carubv_10003900m |
| *Capsella_ru IND* | *Capsella rubella* | Brassicaceae | Carub_v10003163m |
| *Boechera_st HEC3* | *Boechera stricta* | Brassicaceae | Bostr_13129s0018.1 |
| *Boechera_st IND* | *Boechera stricta* | Brassicaceae | Bostr_14547s0021.1 |
| *Brassica_ra HEC3* | *Brassica rapa* | Brassicaceae | Brara_B00326.1 |
| *Brassica_ra IND* | *Brassica rapa* | Brassicaceae | Brara_C02917.1 |
| *Eutrema_sa HEC3* | *Eutrema salsugineum* | Brassicaceae | Thhal_v10015330m |
| *Eutrema_sa IND* | *Eutrema salsugineum* | Brassicaceae | Thhal_v10029526m |
| *Aquilegia_co* 010_00306.1 | *Aquilegia coerulea* | Ranunculacea | Aquco_010_00306.1 |
| *Aquilegia_co* 007_00665.1 | *Aquilegia coerulea* | Ranunculacea | Aquco_007_00665.1 |
| *Aquilegia_co* 022_00317.1 | *Aquilegia coerulea* | Ranunculacea | Aquco_022_00317.1 |
| *Elaeis_gu* XP_010911550.1 | *Elaeis guineensis* | Arecaceae | XP_010911550.1 |
| *Elaeis_gu* XP_010911852.1 | *Elaeis guineensis* | Arecaceae | XP_010911852.1 |
| *Zea_ma* GRMZM5G818776 | *Zea mays* | Poaceae | GRMZM5G818776 |
| *Zea_ma* GRMZM5G812883 | *Zea mays* | Poaceae | GRMZM5G812883 |
| *Zea_ma* GRMZM2G354618 | *Zea mays* | Poaceae | GRMZM2G354618 |
| *Zea_ma* GRMZM5G817854 | *Zea mays* | Poaceae | GRMZM5G817854 |
| *Oryza_sa* Os08g01700.1 | *Oryza sativa* | Poaceae | LOC_Os08g01700.1 |
| *Amborella_tr* scaffold00071.86 | *Amborella trichopoda* | Amborellaceae | AmbTr_scaffold00071.86 |
| *Amborella_tr* scaffold00008.223 | *Amborella trichopoda* | Amborellaceae | AmbTr_scaffold00008.223 |
| *Falcatifolium_ta*  PLYX-2077541 | *Falcatifolium taxoides* | Podocarpaceae | PLYX-2077541 |
| *Halocarpus_bi*  OWFC-2053975 | *Halocarpus bidwillii* | Podocarpaceae | OWFC-2053975 |
| *Torreya_ta* EFMS-2079283 | *Torreya taxifolia* | Taxaceae | EFMS-2079283 |
| *Wollemia_no* RSCE-2008289 | *Wollemia nobilis* | Araucariaceae | RSCE-2008289 |
| *Selaginella_mo* 405220 | *Selaginella moellendorffii* | Selaginellaceae | Selmo_405220 |
| *Physcomitrella_pa* Pp3c3_28970 | *Physcomitrella patens* | Funariaceae | Pp3c3_28970 |
| *Physcomitrella_pa* Pp3c10_19020 | *Physcomitrella patens* | Funariaceae | Pp3c10_19020 |
| *Physcomitrella_pa* Pp3c4_4040 | *Physcomitrella patens* | Funariaceae | Pp3c4_4040 |
| *Physcomitrella_pa* Pp3c17_15380 | *Physcomitrella patens* | Funariaceae | Pp3c17_15380 |
| *Physcomitrella_pa* Pp3c1_40040 | *Physcomitrella patens* | Funariaceae | Pp3c1_40040 |
