# Supplementary material for: CsIVP functions in vasculature development and downy mildew resistance in cucumber
Source: PLoS Biol. 2020 Mar 23;18(3):e3000671. doi: 10.1371/journal.pbio.3000671 (PMC7117775; doi:10.1371/journal.pbio.3000671)
Supplement: S1 Raw Images — (PDF) [file pbio.3000671.s023.pdf]

Fig 2C

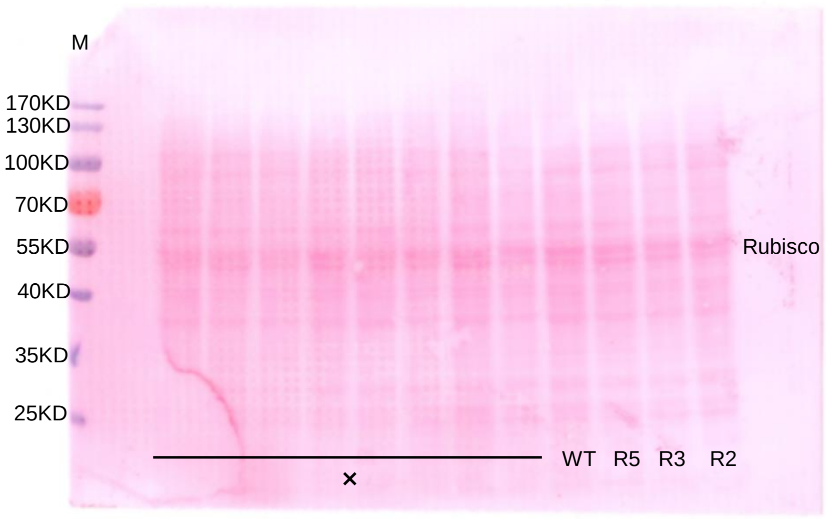

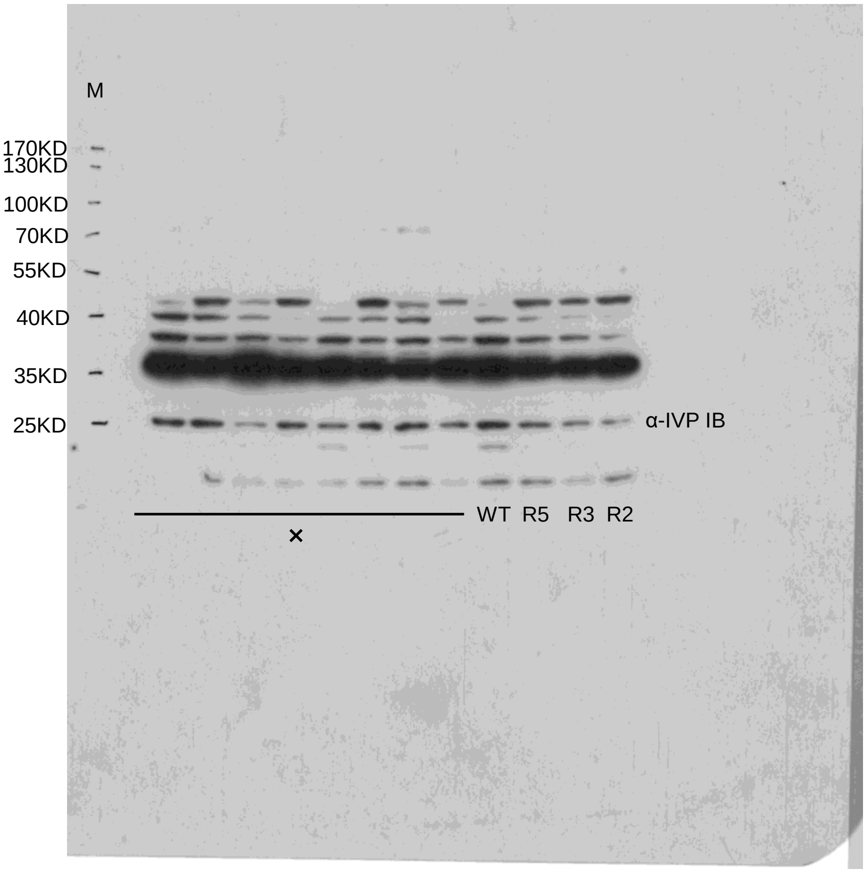

Immunoblots of transgenic lines R5, R3, and R2.

Fig 3D

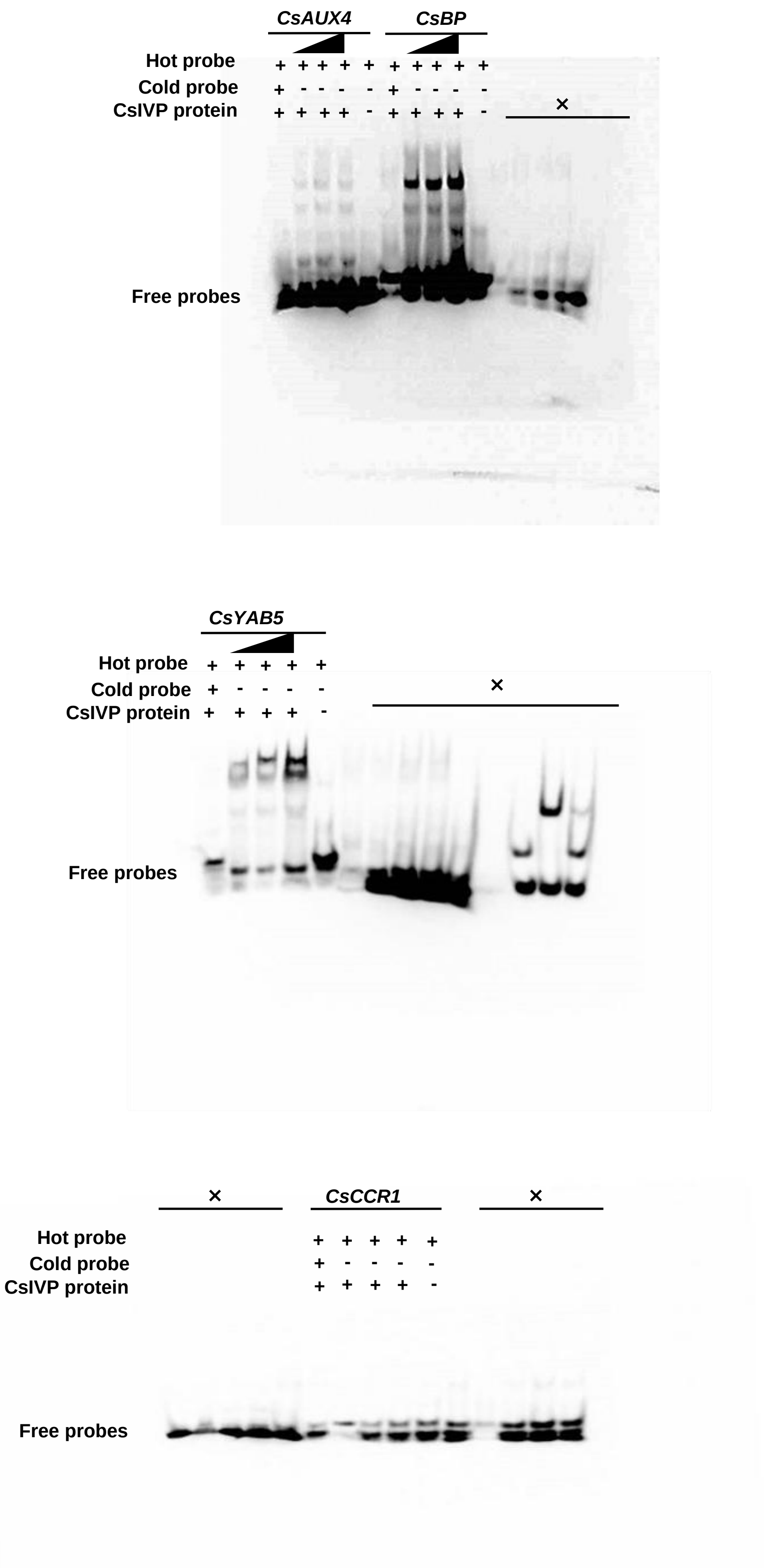

Visualization of direct binding of CsIVP to promoters of *CsYAB5*, *CsBP*, *CsAUX4* and *CsCCR1* via electrophoretic mobility-shift assays.

Fig S6D

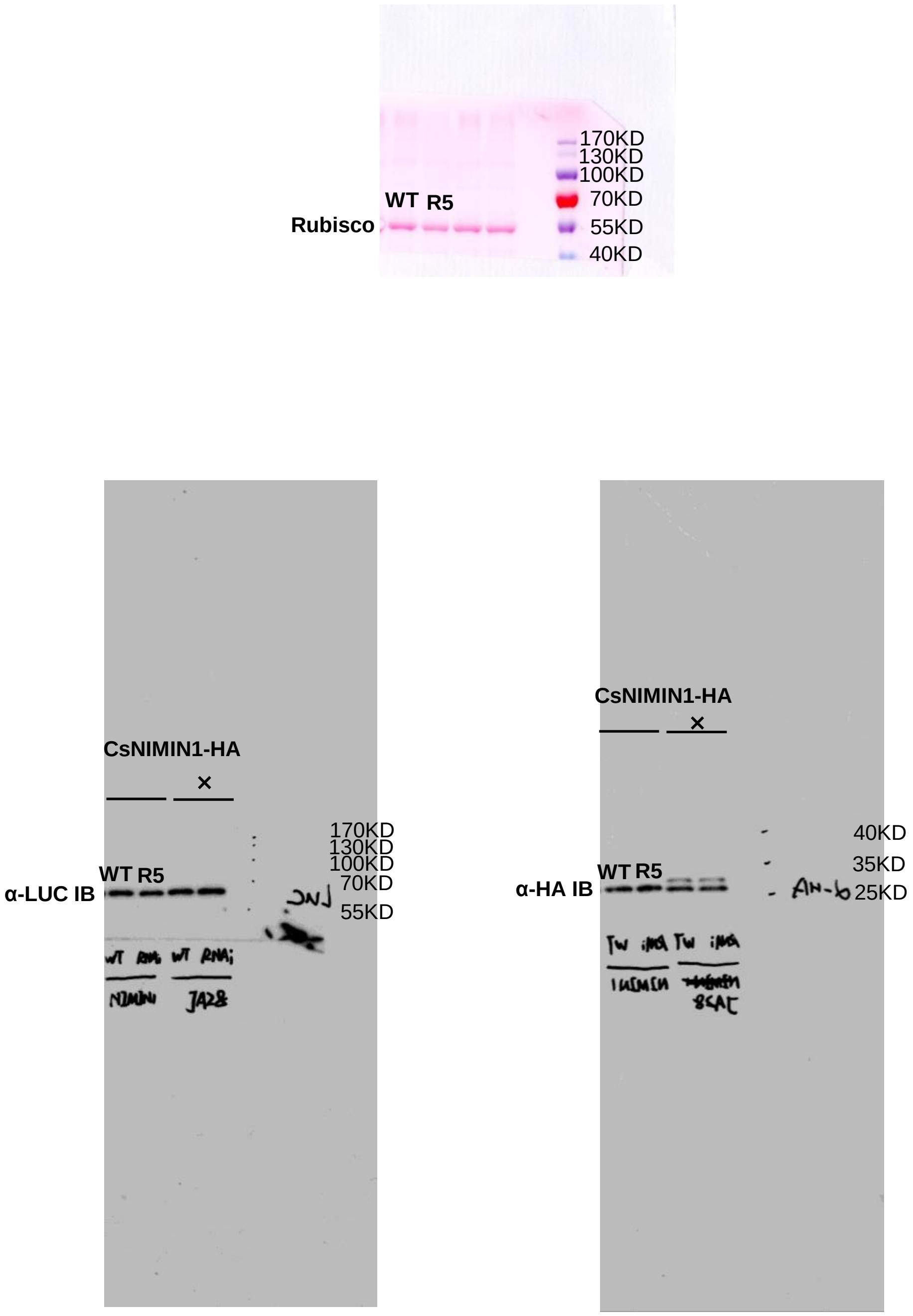

Immunoblots in protoplasts of WT and R5 transgenic plants that overexpressing CsNIMIN1-HA.
